# Non‐Destructive Hydrophobic Engineering of Inverse Catalysts for Methanol Synthesis from CO_2_

**DOI:** 10.1002/advs.202523915

**Published:** 2026-02-15

**Authors:** Dingran Wang, Bingyan Sun, Keran Wang, Di Fu, Ting Fan, Yanghui Lu, Tianfu Zhang, Kaige Wang

**Affiliations:** ^1^ State Key Laboratory of Clean Energy Utilization Zhejiang University Hangzhou P. R. China; ^2^ Low Carbon Research Center State Power Investment Corporation Research Institute Beijing P. R. China

**Keywords:** inverse catalyst, methanol synthesis, physical hydrophobic regulation, ZrO_2_/Cu‐PDVB

## Abstract

CO_2_ hydrogenation to green methanol using renewable hydrogen offers a promising approach for achieving a sustainable carbon cycle. Among various catalyst designs, inverse catalysts have attracted growing interest due to their unique structural advantages. However, the performance of inverse catalysts, such as ZrO_2_/Cu, is hindered by their hydrophilic nature, while the systematic investigations into their surface wettability remain rare. In this study, we report a non‐destructive hydrophobic modification strategy for ZrO_2_/Cu catalyst through physical mixing with polydivinylbenzene (PDVB). The optimized ZrO_2_/Cu‐PDVB (1:1 mass ratio) catalyst achieves a methanol space‐time yield of 920.10 mg_CH3OH_ g_cat_^−^^1^ h^−^^1^ under mild conditions, outperforming the unmodified catalyst by 30%. Additionally, the optimized catalyst also demonstrates outstanding 200 h thermal stability. In situ DRIFTS and related analyses reveal that the PDVB effectively promotes water desorption and diffusion, alleviating its negative impact on the rate‐determining step of formate hydrogenation. This also preserves the size, metallic state of Cu particles, and the abundance of oxygen vacancies, crucial for maintaining the active ZrO_x_‐Cu interface. This work presents a simple, scalable method for adjusting the local microenvironment of inverse catalysts, highlighting the critical yet underexplored role of hydrophobic surface engineering in optimizing water‐sensitive catalytic systems.

## Graphical abstract

In this study, we report a non‐destructive hydrophobic modification strategy for ZrO_2_/Cu catalyst through physical mixing with PDVB. The optimized ZrO_2_/Cu‐PDVB catalyst achieves a methanol space‐time yield of 920.10 mg_CH3OH_ g_cat_^−^^1^ h^−^^1^ under mild conditions, outperforming the unmodified catalyst by 30 %. Additionally, the optimized catalyst also demonstrates outstanding 200 h thermal stability. The systematic characterizations deeply elucidate the internal mechanism.

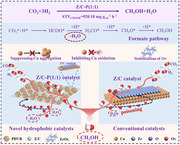

## 1 Introduction

Over the past few decades, the exponential rise in fossil‐based CO_2_ emissions has severely impacted environmental systems, drawing global attention to carbon mitigation [1, 2]. Among the most promising strategies to alleviate the adverse effects of excessive CO_2_ emissions is the catalytic conversion of CO_2_ into value‐added fuels and chemicals, thereby enabling carbon‐neutral processes [3, 4, 5]. Methanol, serving as both a clean transportation fuel and a basic chemical, possesses excellent developmental potential [6, 7]. Consequently, the sustainable production of green methanol from CO_2_ and renewable hydrogen offers a viable pathway to achieving a carbon‐closed loop and net‐zero emissions, contributing to the global transition toward sustainable chemical manufacturing.

A major challenge in this strategy lies in the development of efficient catalysts, which has attracted significant research interest. Currently, copper‐based catalysts are the most predominantly utilized for the hydrogenation of CO_2_ to methanol, due to their superior catalytic activity and cost‐effectiveness [8, 9, 10, 11]. However, conventional Cu/oxide catalysts suffered from overly stabilized formate intermediates and hindered hydrogen transfer due to strong CO_2_ adsorption on oxide supports, leading to poor low‐temperature activity [12, 13, 14]. Moreover, limited thermal stability caused by Cu sintering under harsh conditions further constrains their catalytic performance [15, 16]. To overcome these challenges, inverse interface engineering has also proven effective [17]. Inverse catalysts, consisting of oxide nano‐islands supported on metallic material, have demonstrated improved hydrogenation activity under mild reaction conditions [12]. It has been reported that the inverse ZrO_2_/Cu catalyst with a ZrO_2_‐Cu interface significantly enhanced methanol formation, achieving a methanol space‐time yield (STY_CH3OH_) of 524 mg_CH3OH_ g_cat_^−1^ h^−1^ at 220°C, 3.3 times higher than conventional Cu/ZrO_2_ catalysts [5]. Further studies have shown that increasing the Cu/Zr ratio and adjusting the size of Cu particles led to improved methanol productivity [2, 18]. Additionally, in ZnO/Cu systems, dispersing ZnO species on Cu surfaces enhanced CO_2_ activation and methanol formation [19]. The introduction of oxygen vacancies (O_v_) at the ZnO_1‐x_/Cu interface further improves performance by facilitating CO_2_ activation and increasing methanol yield [20]. Despite significant advancements have been made in enhancing catalytic performance, most inverse catalysts were hydrophilic, and their performance still required further improvement.

CO_2_ hydrogenation to methanol is a reaction that is severely limited by water, both thermodynamically and kinetically. Previous studies have demonstrated that the selective and rapid water removal from the catalytic surface's microenvironment effectively mitigated these adverse effects [21, 22, 23, 24]. Therefore, exploring hydrophobic modification is of great significance for further enhancing the catalytic performance. Most hydrophobic modification strategies currently rely on chemical approaches, such as self‐assembled monolayers or organosilane treatments. However, the macromolecules introduced during chemical modification can alter the catalyst's intrinsic surface structure and block active sites, potentially reducing catalytic activity [25, 26, 27]. In contrast, physical modification strategies enable the rapid diffusion of water from an unchanged catalyst surface, offering a simpler, more scalable, and non‐destructive preparation process compared to membrane reactors, thereby emerging as a highly promising alternative. For instance, in CO and CO_2_‐based reactions, the physical mixing of PDVB with oxide‐supported catalysts was reported to enhance light olefin or methanol productivity without altering the catalyst's intrinsic surface properties [25, 26, 28, 29]. The above reports indicated that employing PDVB for hydrophobic modification is a highly promising strategy. However, the previous studies have exclusively focused on the conventional catalysts with metal supported on metal oxides. Inverse catalysts, such as ZrO_2_/Cu, possess unique interfacial structures that are inherently water‐sensitive, necessitating a distinct understanding of how hydrophobic regulation affects their complex ZrO_x_‐Cu interfaces and specific reaction intermediates. To date, no investigation has explored the application of PDVB in inverse catalyst configurations, particularly focusing on how hydrophobic modification can mitigate water‐induced deactivation at the continuous metal‐oxide interface. The mechanism underlying this modification in inverse systems remains largely unexplored.

Here, we have innovatively synthesized a hydrophobic ZrO_2_/Cu‐PDVB catalyst through a physical modification strategy. Distinct from conventional supported systems, this study investigates the microscopic influence of a hydrophobic microenvironment on the interfacial mechanisms of inverse catalysts (Figure S1). Under typical reaction conditions of 240°C, the catalyst achieved a STY_CH3OH_ of 920.10 mg_CH3OH_ g_cat_^−1^ h^−1^ and demonstrated outstanding stability over a 200 h testing period. In direct comparison with the unmodified inverse catalyst, this represents about 30% performance enhancement, achieved without altering the catalyst's intrinsic composition or active‐site structure. The comprehensive results of in situ DRIFTS, H_2_O‐TPD‐MS, CO_2_‐TPD, and several other characterizations indicated that this physical modification strategy, while achieving non‐destructive modification, facilitated the rapid desorption and diffusion of water on the catalyst surface. This ensured the efficient transformation of formate intermediates to methoxy groups, ultimately leading to a significant enhancement in catalytic performance. This work pioneers a non‐invasive, scalable approach to regulate reaction microenvironments, offering a universal solution for water‐sensitive catalytic processes.

## 2 Experimental

### 2.1 Chemicals and Materials

Cu(NO_3_)_2_⋅3H_2_O (99 wt%), Zr(NO_3_)_4_⋅5H_2_O (99 wt%), and ethanol were obtained by Sinopharm Chemical Reagent Co., Ltd. Oxalic Acid (99 wt%), divinylbenzene (DVB), azobisisobutyronitrile (AIBN), and SiO_2_ were purchased from Aladdin Chemical Reagent Company. Methanol, carbon nanotubes (CNTs), polytetrafluoroethylene (PTFE), and polyacrylonitrile (PAN) were supplied from Macklin. All materials were used without further purification.

### 2.2 Catalyst Preparation

#### 2.2.1 Synthesis of ZrO_2_/Cu‐0.1 Inverse Catalysts

The ZrO_2_/Cu‐0.1(where x is the atomic percentage of ZrO_2_ in the sample) was synthesized by a two‐step oxalate‐mediated precipitation (CP) method. The oxalic acid was used as a precipitating agent. 0.02 mol of Cu(NO_3_)_2_·3H_2_O was dissolved in 80 mL of ethanol, designated as solution A, while a certain quantity of oxalic acid was dissolved in 100 mL of ethanol, designated as solution B. Subsequently, solution A was added dropwise into solution B with a peristaltic pump under vigorous stirring. After 30 min reaction, the Zr(NO_3_)_4_⋅5H_2_O (0.002 mol) ethanol solution and 20 mL of 0.5 m oxalic acid ethanol solution were added to the above mixed solution. After 4 h, the resultant solid was separated by centrifugation, followed by washing with ethanol and drying at 60°C overnight. The obtained powder was calcined in the furnace at 400°C for 3 h.

#### 2.2.2 Synthesis of Nonporous PDVB

Nonporous polydivinylbenzene (PDVB) was synthesized by dissolving 1 g of azobisisobutyronitrile (AIBN) in 20 g of divinylbenzene (DVB) under continuous stirring at room temperature for 1 h. The resulting mixture was then transferred to a Teflon‐lined stainless‐steel autoclave and subjected to thermal polymerization at 100°C for 24 h. The obtained solid product was washed thoroughly with methanol and dried at 100°C overnight.

### 2.3 Catalyst Characterizations

The water droplet contact angle (CA) measurements for all catalysts were performed using a Dataphysics OCA 20 system. The catalysts were pressed into flat pellets, and 1 µL water droplets were deposited. Contact angles were then determined via image analysis. The crystallinity of copper and zirconia nanoparticles was researched by powder X‐ray diffraction (XRD). The analysis utilized Cu Kα radiation (λ = 1.542Å), operating at 40 kV and 40 mA, with a scanning rate of 5 ° per minute.

Transmission electron microscopy (TEM), high‐resolution TEM (HRTEM), and energy dispersive X‐ray spectrometry (EDS) were performed on a FEI Talos F200S System at 200 kV. The precise elemental composition of the catalyst was determined by inductively coupled plasma optical emission spectrometry (ICP‐OES) using an IRIS Intrepid II XSP instrument.

The textural properties of the catalysts were analyzed via N_2_ adsorption–desorption isotherms using a Micromeritics ASAP 2020 surface area and porosity analyzer. The specific surface areas were calculated using the Brunauer‐Emmett‐Teller (BET) method, while the pore size distribution was evaluated based on the desorption branch of the Barrett‐Joyner‐Halenda (BJH) model.

Thermogravimetry analysis and differential scanning calorimetry (TGA‐DSC) were performed on an LCD200M (TILON) instrument under a flowing air atmosphere with a heating rate of 10 °C/min. X‐ray photoelectron spectra (XPS) were obtained using a Thermo Scientific Nexsa equipped with an Al Kα X‐ray source (wavelength 1486.6 eV) to analyze the valence states of surface elements. The energy scan range was from 0 to 1200 eV, and the scanning results were calibrated using the C 1s peak.

CO_2_‐temperature‐programmed desorption (CO_2_‐TPD) measurements were performed on a BELCAT‐B instrument. The catalyst was reduced in pure H_2_ at 300°C for 120 min (5°C min^−^^1^), purged with Ar for 30 min, and cooled to 60°C. It was then exposed to 10% CO_2_/Ar for 60 min, followed by Ar purging for 40 min to remove non‐adsorbed CO_2_. After baseline stabilization, CO_2_ desorption was recorded by heating from room temperature to 700°C at 10°C min^−^^1^.

TPR and N_2_O titration experiments were obtained using a Hiden DECRA. 30–50 mg sample was loaded in a U‐shaped quartz reactor. The sample was heated to 150°C in Ar (30 mL min^−^^1^) at 10°C min^−^^1^ and held for 30 min to remove surface adsorbates. After cooling to 50°C, the gas was switched to 10% H_2_/Ar (30 mL min^−^^1^), and the catalyst was heated to 300°C at 10°C min^−^^1^, then held for 120 min. The flow was returned to Ar (30 mL min^−^^1^) and the reactor was cooled to 50°C. At 50°C, 10% N_2_O/Ar (30 mL min^−^^1^) was introduced for 30 min to perform the surface reaction, followed by a He purge for 30 min. The gas was switched to 10% H_2_/Ar (30 mL min^−^^1^), and the sample was heated to 300°C at 10°C min^−^^1^ and reduced for 15 min. The hydrogen consumption during the first TPR was denoted as X, and the hydrogen consumption during the second TPR was denoted as Y.

^13^C nuclear magnetic resonance (NMR) was conducted on a Bruker Avance 600 AV. FT‐IR spectra were recorded on a Thermo IS50. The Electron Paramagnetic Resonance (EPR) experiments were carried out on a Bruker EMX spectrometer at room temperature, with a microwave frequency of 9.8 GHz.

H_2_O temperature‐programmed desorption (H_2_O‐TPD‐mass) tests were performed on a Microtrac BELCatIIJapan. The sample was reduced with 10.0 vol% H_2_ (40 mL/min) at 300°C for 1 h, then cooled to 30°C in pure He. H_2_O/He was introduced until saturation, and the sample was heated at 5°C/min to 350°C, with desorbed H_2_O detected by mass spectrometry.

The vapor adsorption‐desorption isotherm was measured at 313 K using a Microtrac BELCat II instrument in Japan. Prior to the adsorption test, the catalyst was vacuum‐degassed at 200°C for 2 h.

In situ diffuse reflectance infrared Fourier transform spectroscopy (DRIFTS) analysis was conducted at 240°C using an infrared spectrometer (Thermo, Nicolet Is50) equipped with an MCT detector. The sample was reduced in H_2_ (40 mL/min) at 300°C for 3 h, followed by a 30‐min purge with He. Subsequently, a gas mixture of H_2_/CO_2_/Ar with a volume ratio of 72/24/4 vol% was introduced at a flow rate of 30 mL/min, and in situ DRIFT spectra were recorded. For comparison, deionized water (3 vol%) was introduced via bubbling, and the spectral changes were recorded in real‐time. The spectrum of the reduced catalyst in Ar flow at the reaction temperature was collected as the background.

### 2.4 Catalytic Evaluation and Product Analysis

Typically, 200 mg of catalyst was fixed in the isothermal zone of a fixed‐bed quartz reactor using quartz wool. The multi‐channel fixed‐bed reactor is depicted in Figure S2. Prior to the reaction, the catalyst was pre‐reduced in pure H_2_ (40 mL/min) at 300°C for 3 h with a heating rate of 5°C/min. The temperature was then adjusted to the desired reaction temperature, and the gas feed was switched to H_2_/CO_2_/Ar with a volume ratio of 72/24/4 vol%. Reactions were conducted at 5 MPa, and reactants and products were analyzed using an online gas chromatograph equipped with a thermal conductivity detector (TCD) and a flame ionization detector (FID). To prevent methanol condensation, the reactor‐to‐gas chromatograph line was maintained at 140°C. The gas hourly space velocity (GHSV) was calculated based on the mass of the ZrO_2_/Cu catalyst, excluding the PDVB component. CO_2_ conversion(Conv_C_O_2_), product selectivity(Sel _product_), and methanol space‐time yield (STY_CH3OH_) were determined using the following formulas (Equations (1), (2), (3)). The variable n represented the mole rate (mol/h).

(1) $$\mathrm{Con}v_{CO_{2}}=\frac{n_{\mathrm{in}(CO_{2})}\;-\;n_{\mathrm{out}(CO_{2})}}{n_{\mathrm{in}(CO_{2})}}\;\times \;100\%$$

(2) $$\mathrm{Se}l_{CH_{3}\mathrm{OH}}=\frac{n_{\mathrm{out}\left(CH_{3}\mathrm{OH}\right)}}{n_{\mathrm{out}\left(CH_{3}\mathrm{OH}\right)}+n_{\mathrm{out}\left(CH_{4}\right)}+n_{\mathrm{out}\left(\mathrm{CO}\right)}}\;\times \;100\%$$

(3)

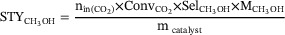




## 3 Results and Discussion

### 3.1 Catalysts Screening

**FIGURE 1:**
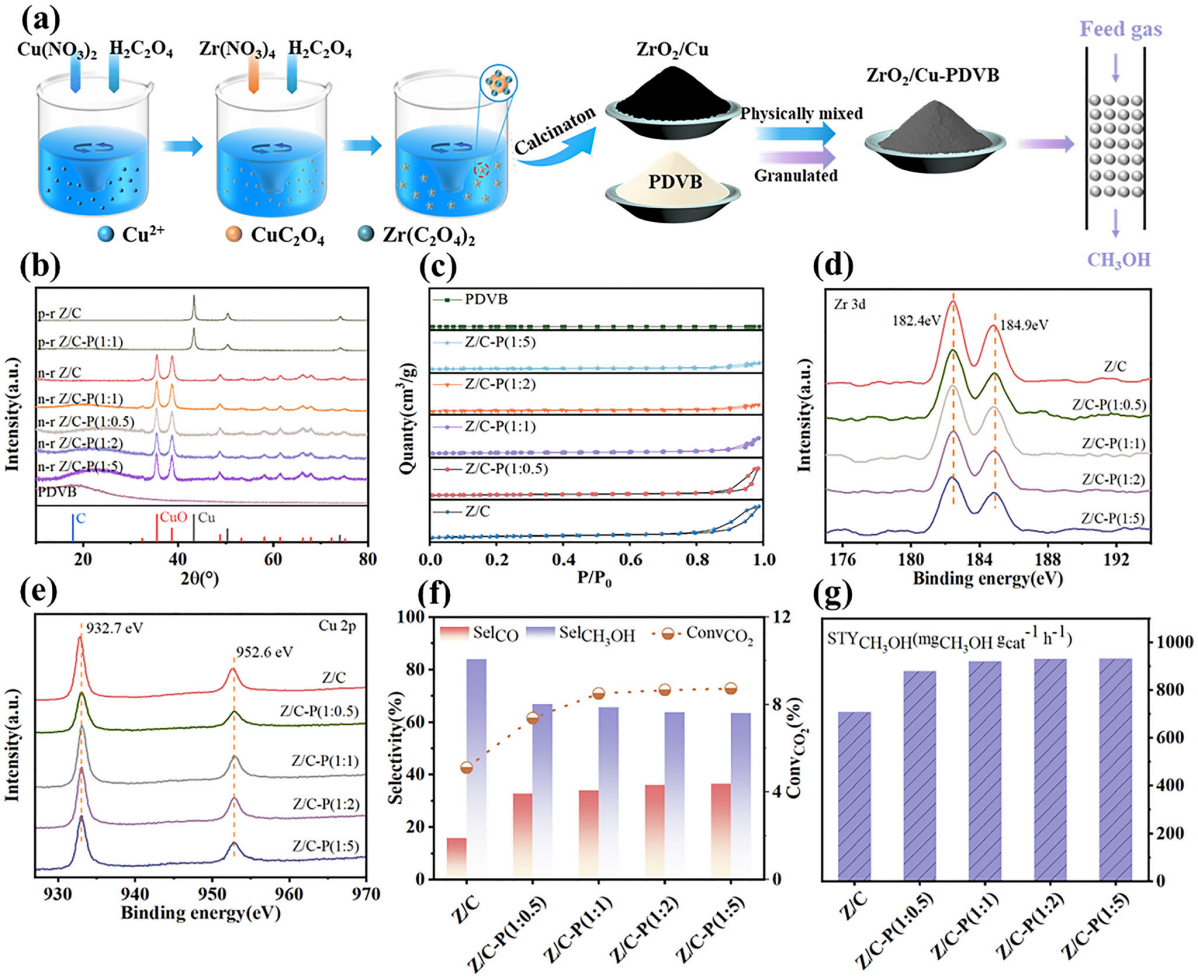
Catalyst preparation, characterization, and performance evaluation. (a) Schematic diagram of catalyst preparation and performance testing. (b) XRD patterns and (c) N_2_ adsorption‐desorption isotherms of catalysts with varying mass ratios. The XPS spectra of the reduced catalysts: (d) Zr 3d and (e) Cu 2p. (f) and (g) Performance of the catalysts at 240°C, 5 MPa, and 48,000 mL g_cat_^−^^1^ h^−^^1^.

In this section, to investigate the effect of hydrophobic modification on the CO_2_ hydrogenation performance of inverse ZrO_2_/Cu catalysts, a series of ZrO_2_/Cu‐PDVB (1:x) catalysts were synthesized, where x represented the mass ratio of PDVB to ZrO_2_/Cu and took values of 0, 0.5, 1, 2, and 5. The original catalyst without PDVB was denoted as Z/C, while those with varying PDVB contents were labeled as Z/C‐P (1:x). The overall experimental procedure was illustrated in Figure 1a, and the photographs of Z/C, PDVB, and their mixture were shown in Figure S3, demonstrating the distinct appearances of each component and the homogeneous morphology after mixing. Z/C was prepared via a two‐step oxalate co‐precipitation method, followed by physical mixing with PDVB to obtain the target catalysts. The physical mixing involved blending Z/C and PDVB powders and subsequently granulating. Finally, the catalytic performance was assessed in a multi‐channel fixed‐bed reactor. As shown in Figure 1b, XRD results reveal that the calcined and reduced Z/C samples exhibit characteristic peaks corresponding to CuO and Cu, respectively [2, 5, 18, 30]. Specifically, the pre‐reduced catalysts were denoted as p‐r catalysts, while the non‐reduced catalysts were denoted as n‐r catalysts. The physically mixed Z/C‐P samples also displayed the same metallic signatures, accompanied by a broad diffraction peak around 20 °, attributed to the presence of amorphous PDVB [28]. With increasing amounts of PDVB, the intensity of the carbon signal gradually rose, indicating the successful incorporation of a quantifiable amount of PDVB via the physical regulation strategy [26]. No characteristic peaks corresponding to ZrO_2_ were observed in the diffraction profiles of any of the catalysts, suggesting that the ZrO_2_ species obtained through the oxalate co‐precipitation method were likely in an amorphous state, consistent with previous literature reports [2, 5, 31]. Nevertheless, the ICP analysis (Table S1) reveals that both the original Z/C and the modified Z/C‐P (1:1) catalysts exhibit a Zr:Cu molar ratio of 1:10, aligning with the theoretical design specifications. Collectively, these results confirmed that the catalysts were successfully synthesized. Given that the PDVB employed in this study was essentially non‐porous (BET surface area < 5 m^2^ g^−^^1^), the overall specific surface area of the composites decreased progressively as the PDVB loading increased (Figure 1c) [25, 26, 28]. Figure 1d,e display the high‐resolution XPS spectra of Zr 3d and Cu 2p regions, respectively [32, 33, 34, 35]. The binding energies of both elements remained essentially constant across all samples, indicating that the introduction of PDVB via physical mixing did not alter the electronic states of Zr or Cu species. This confirmed the absence of chemical bonding or charge transfer between PDVB and ZrO_2_/Cu, validating the non‐invasive nature of the modification strategy. The physical mixing approach thus ensured that the active metal‐oxide interface remained structurally and electronically intact. These analysis confirmed that the fundamental structure and composition of the Z/C catalyst remained unchanged after physical mixing with PDVB.

**FIGURE 2:**
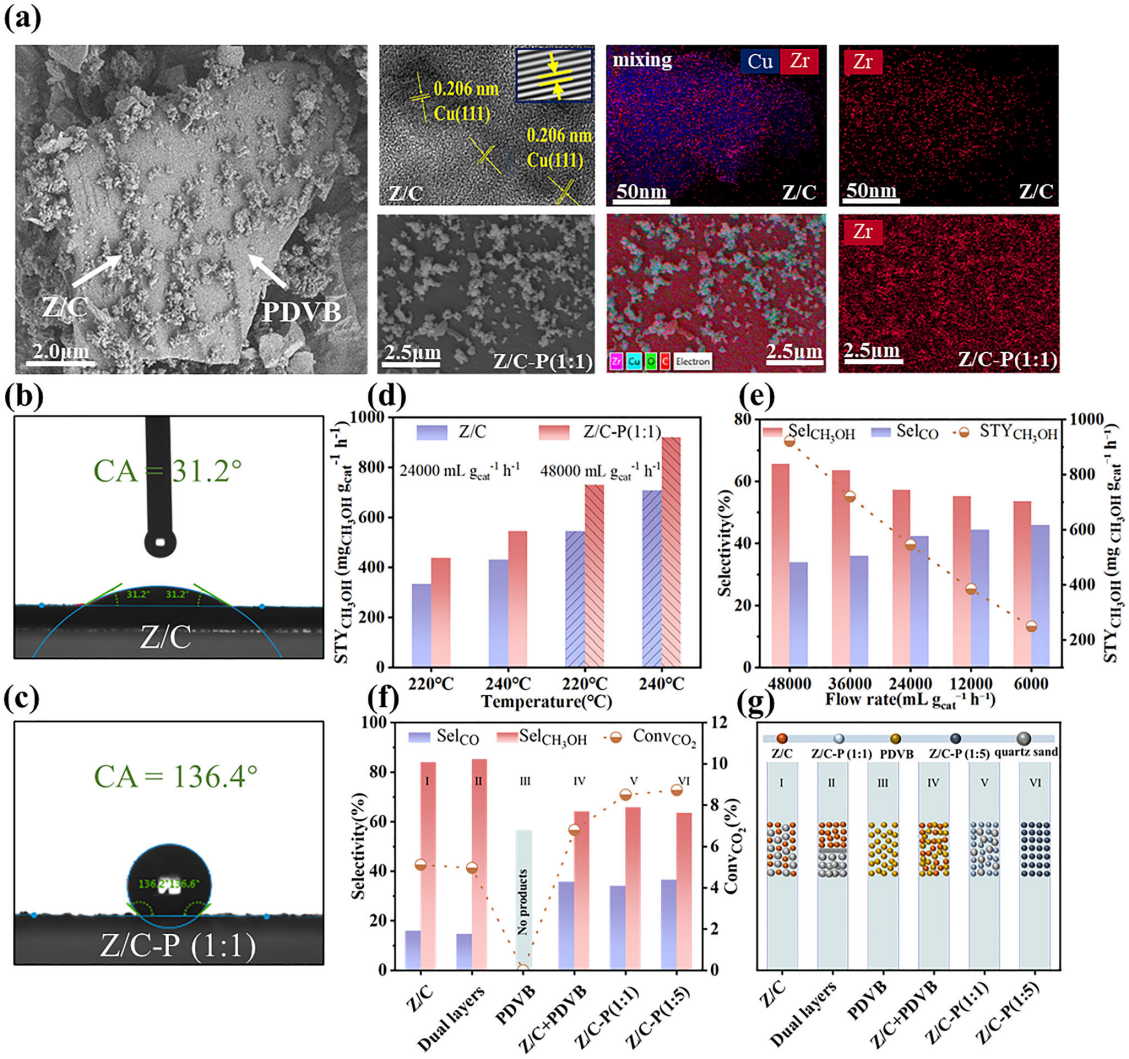
Characterization and performance comparison of Z/C and Z/C‐P (1:1) catalysts. (a) SEM, HRTEM, and EDS mapping images of Z/C and Z/C‐P (1:1) catalysts. (b) Water droplet contact angle (CA) of Z/C catalyst. (c) Water droplet contact angle (CA) of Z/C‐P (1:1) catalyst. (d) Catalytic performance of Z/C and Z/C‐P (1:1) catalysts at different temperatures and GHSV. (e) Catalytic performance of the Z/C‐P (1:1) catalyst at 240°C and 48 000 mL g_cat_^−^^1^ h^−^^1^ with varying GHSV. (f) Effect of different mixing methods on catalyst performance. (g) Schematic diagram of the corresponding mixing methods.

CO_2_ hydrogenation was conducted at 240°C, 5 MPa and 48 000 mL g_cat_^−1^ h^−1^. As shown in Figure 1f,g and Tables S2 and S3, the promoting effect becomes more pronounced with increasing PDVB content. However, beyond the ratio of 1:1, further addition of PDVB resulted in negligible changes in catalytic performance. Therefore, the Z/C‐P (1:1) catalyst was selected as the optimal catalyst for subsequent mechanistic and kinetic investigations (series proof shown in Figure 2). The Z/C‐P (1:1) catalyst achieved a CO_2_ conversion of 8.50% and a methanol space‐time yield (STY_CH3OH_) of 920.10 mg_CH3OH_ g_cat_^−^^1^ h^−^^1^, representing an approximate 30.00% improvement over the unmodified Z/C, which showed a lower CO_2_ conversion of 5.11% and a STY_CH3OH_ of 707.27 mg_CH3OH_ g_cat_^−^^1^ h^−^^1^. The enhanced performance was attributed to the introduction of hydrophobic PDVB, which facilitated the rapid desorption of surface water molecules formed during the reaction, thereby shifting the equilibrium of adsorbed water (^*^H_2_O ⇌ ^*^ + H_2_O, where ^*^ denotes an active surface site) and freeing up active sites for continuous CO_2_ activation. The hydrophobic interface constructed by PDVB facilitated prompt water desorption and diffusion, effectively lowering water activity and alleviating site inhibition. While this enhanced CO_2_ conversion and methanol yield, a decline in methanol selectivity was observed. Thermodynamically, this was attributed to the differential sensitivity of competing reactions to water removal. As demonstrated by literature [36, 37, 38, 39], the RWGS reaction was more sensitive to reduced water concentration than the methanol pathway. Consequently, the rapid water removal exerted a more positive effect on the RWGS equilibrium, disproportionately increasing the carbon flux toward CO. Additionally, methanol formation consumed three equivalents of H_2_ compared to one for RWGS. Therefore, the high conversion at the hydrophobic interface led to localized H_2_ depletion, further favoring the hydrogen‐lean RWGS pathway. Kinetically, despite this thermodynamic selectivity‐productivity trade‐off, the enhanced methanol space‐time yield (STY) was achieved by accelerating the rate‐determining step of HCOO^*^ hydrogenation to CH_3_O^*^, as confirmed by in situ DRIFTS. And in industrial production, the generated CO can be utilized for methanol synthesis through tail gas recycling. The overall efficiency can be further optimized by adjusting the GHSV or operating other conditions to favor the kinetic regime of methanol formation.

### 3.2 Structural Characterization and Catalytic Performance of the Z/C‐P (1:1) Catalyst

The morphology of the Z/C‐P (1:1) catalyst was characterized by SEM. As shown in Figure 2a, after physical mixing with PDVB, the Z/C catalyst is partially distributed on the surface of the larger PDVB particles, while the remaining portion is dispersed within the interstitial spaces between different PDVB domains (Figure S4) [28]. High‐resolution TEM images of the Z/C‐P (1:1) catalyst revealed exposed Cu(111) lattice planes with an interplanar spacing of 0.206 nm, unequivocally confirming the reduction of the Cu species [40, 41, 42]. EDS mapping for both types of catalysts indicates a homogeneous elemental distribution without evident aggregation (Figure S5), suggesting that the physical mixing with PDVB does not alter the original elemental dispersion of the catalyst. In the EDS maps, signals corresponding to Cu, Zr, and O originate from the Z/C component, while the carbon signal was attributable to PDVB [28]. Notably, Zr was uniformly distributed across the Cu surface, indicating that the synthesized catalyst maintained a favorable inverse configuration. To further elucidate the superior performance of the Z/C‐P (1:1) catalyst, we investigated its surface wettability. The pristine Z/C catalyst exhibits hydrophilic behavior, as evidenced by a water contact angle of 31.20 ° (Figure 2b). In contrast, the hydrophobic PDVB powder displays a contact angle of 150.10 ° (Figure S6). The composite Z/C‐P (1:1) catalyst shows a significantly increased contact angle of 136.40 ° (Figure 2c), demonstrating pronounced hydrophobicity. In the context of green methanol synthesis, where water generation was a major challenge, the hydrophobic nature of the Z/C‐P (1:1) catalyst facilitated rapid desorption of the water byproduct from the catalyst surface, thereby preserving the activity of the catalytic sites [25, 26, 28].

Temperature and gas feeding rate critically determine methanol productivity in CO_2_ hydrogenation [43, 44, 45]. As shown in Figure S7 and Table S4, the catalytic performance of Z/C‐P catalysts with varying mass ratios at 220 °C, 5 MPa, and 48 000 mL g_cat_^−^^1^ h^−^^1^ reveals a progressive increase in methanol productivity with rising PDVB content. However, once the mass ratio reached 1:1, further addition of PDVB no longer contributed to any significant enhancement in performance—a trend consistent with observations at 240 °C (Figure 1f,g). Nevertheless, all catalysts exhibited lower methanol yields at 220 °C compared to their counterparts operating under identical conditions at 240 °C. Similarly, as depicted in Figure 2d, when the GHSV is adjusted to 24 000 mL g_cat_^−^^1^ h^−^^1^ at 240 °C, the Z/C‐P (1:1) catalyst exhibits a methanol productivity of 545.34 mg _CH3OH_ g_cat_^−^^1^ h^−^^1^ with a CO_2_ conversion rate of 11.56%. These values surpassed those observed at 220 °C, where the corresponding methanol productivity was 438.86 mg _CH3OH_ g_cat_^−^^1^ h^−^^1^. Literature indicated that inverse catalysts generally operated within the low‐temperature regime below 240 °C [18, 46, 47, 48], further validating the selection of 240 °C as the representative reaction temperature. The higher gas flow rates led to more pronounced enhancements in catalytic activity. For instance, at 240 °C, increasing the GHSV from 24 000 to 48 000 mL g_cat_^−^^1^ h^−^^1^ results in a 24.26% and 25.89% improvement in STY_CH3OH_, respectively (Figure 2d). This enhancement may be attributed to the fact that the hydrophobic modification accelerated CO_2_ conversion, while the increase in GHSV enhanced methanol selectivity [26]. To further investigate the influence of gas feeding rates on catalytic performance, a series of experiments was conducted using the Z/C‐P (1:1) catalyst (Figure 2e; Figure S8). As the gas feeding rate increased, the STY_CH3OH_ rose markedly from 248.21 mg_ CH3OH_ g_cat_^−^^1^ h^−^^1^ at 6 000 mL gcat^−^^1^ h^−^^1^ to 920.10 mg_ CH3OH_ g_cat_^−^^1^ h^−^^1^ at 48 000 mL g_cat_^−^^1^ h^−^^1^, accompanied by a rise in methanol selectivity from 53.65% to 65.72%. A similar trend was observed when compared with the unmodified Z/C catalyst, where higher gas feeding rates led to a more pronounced improvement in catalytic performance at the same temperature [2, 5]. Based on the above results, it is evident that PDVB consistently enhances the catalytic performance of Z/C.

To reveal the interaction mechanism of PDVB, the mixing manners of the individual components were systematically varied. In each experiment, the mass of Z/C catalyst was fixed at 0.2 g, and the intrinsic GHSV was kept consistent relative to the amount of Z/C. Specifically, Z/C‐P (1:5) (Group VI) was selected as the reference to ensure uniformity in catalyst bed volume across all tests, with inert quartz sand used to compensate for volume differences. The corresponding catalytic performance was presented in Figure 2f,g and Figure S9. Group I, consisting of Z/C and quartz sand, exhibited a CO_2_ conversion of 5.11% and methanol selectivity of 83.95%, which was closely similar to the dual‐bed configurations. Group III, composed solely of PDVB, displayed no catalytic activity, confirming the chemical inertness of PDVB and reinforcing its role as a hydrophobic modifier rather than a catalytic component. In Group IV, where Z/C and PDVB were pelletized separately and then physically mixed in the reactor bed, the catalyst achieved the CO_2_ conversion and methanol productivity with 6.80% and 717.22 mg_ CH3OH_ g_cat_^−^^1^ h^−^^1^. Remarkably, the powder‐blended catalyst in Group V demonstrated substantially higher values, with 8.50% CO_2_ conversion and 920.10 mg_ CH3OH _g_cat_^−^^1^ h^−^^1^ productivity. These findings suggested that the close proximity and intimate contact between Z/C and PDVB were crucial for achieving effective hydrophobic modification. This observation was consistent with previous studies [25, 26, 28].

### 3.3 Systematic Analysis of the Hydrophobic Mechanism of Z/C‐P (1:1) and PDVB

**FIGURE 3:**
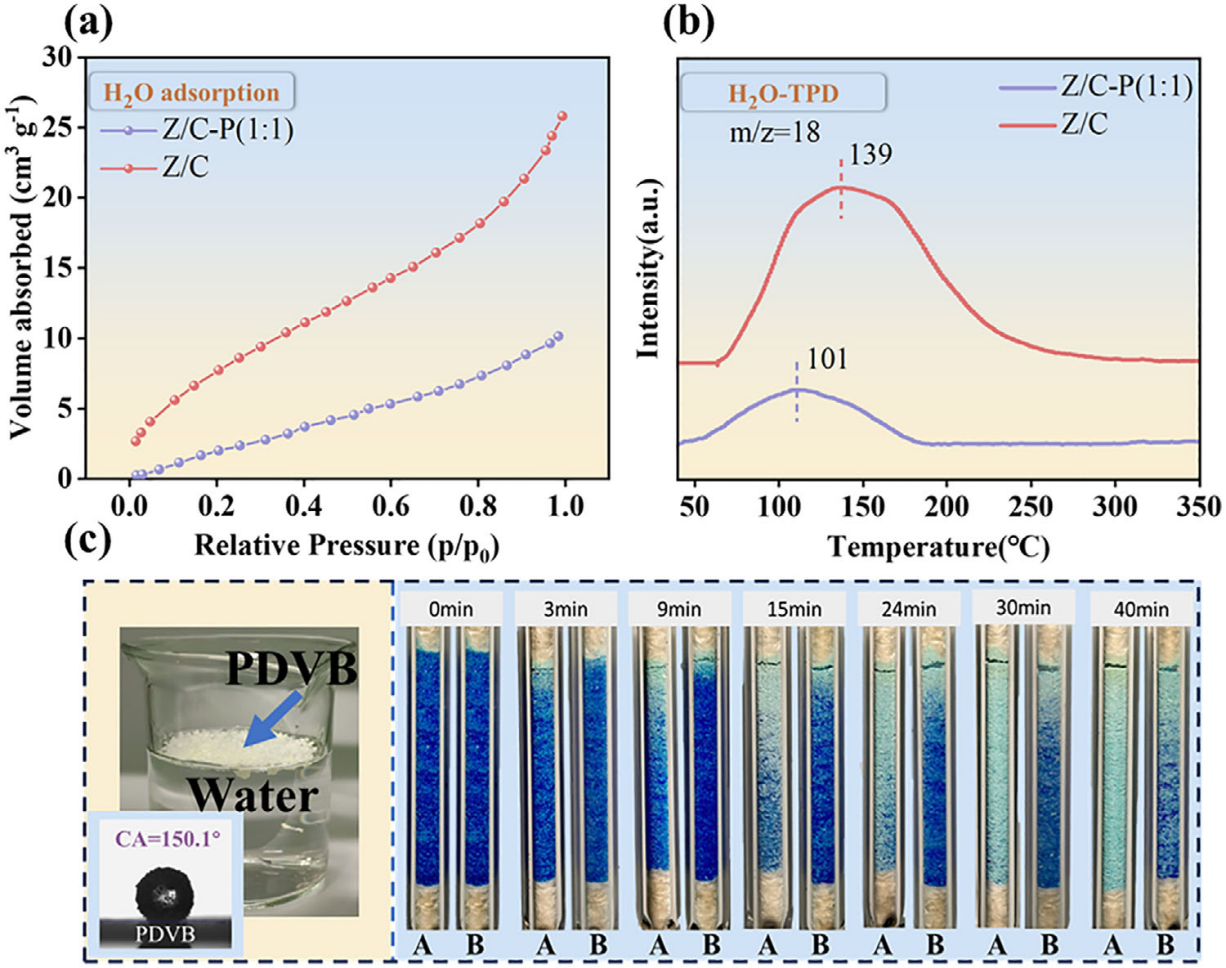
Hydrophobicity characterization of Z/C‐P (1:1) catalyst and PDVB. (a) Water adsorption‐desorption analysis and (b) H_2_O‐TPD‐mass analysis of Z/C and Z/C‐P (1:1) catalysts. (c) Hydrophobicity verification of PDVB. Specifically, 2 g of CuSO_4_·5H_2_O was homogeneously mixed with 2 mg of PDVB and purged with N_2_ at 100 °C at a flow rate of 20 mL min^−^^1^. A represents the presence of PDVB, while B indicates the absence of PDVB.

Based on the contact angle measurements (Figure 2c), the Z/C‐P(1:1) catalyst exhibited significantly enhanced hydrophobicity. We hypothesized that the primary role of the PDVB lied in modulating local dehydration through rapid water adsorption–desorption dynamics. As shown in Figure 3a, the amount of water vapor adsorbed under varying partial pressures was quantified. The total volume of adsorbed water vapor on Z/C and Z/C‐P (1:1) catalysts was 25.78 and 10.14 cm^3^ g^−^^1^, respectively. At every vapor pressure tested, the hydrophilic Z/C catalyst adsorbed more than twice the amount of water compared to the hydrophobic Z/C‐P (1:1) catalyst. These results indicated that the incorporation of PDVB significantly suppressed water adsorption on the catalyst surface, thereby minimizing the negative effects of water on active catalytic sites [28, 49]. In addition, H_2_O‐TPD‐mass was employed to examine the interaction between water molecules and the catalyst surfaces (Figure 3b). The unmodified Z/C catalyst exhibited a significant water desorption peak at 139 °C, while the Z/C‐P (1:1) catalyst showed a shifted peak at 101 °C under identical conditions. This suggested that PDVB modification lowered the adsorption energy of water on the catalyst surface, facilitating faster desorption of adsorbed water [50, 51]. The Z/C catalyst also demonstrated a broader water adsorption region from 59 °C to 310 °C, whereas the Z/C‐P(1:1) catalyst exhibited a much narrower range, further supporting the conclusions presented in Figure 3a [52, 53]. In summary, these results clearly demonstrated that the modified Z/C‐P (1:1) catalyst effectively suppressed water adsorption and promoted the rapid desorption of surface‐adsorbed water, which was crucial for maintaining active catalytic sites in water‐sensitive reactions.

Figure 3c illustrates the inherent hydrophobic nature of PDVB. When PDVB was placed in a beaker containing deionized water, it was observed to be virtually incompatible with the water, and no visible microdroplets were physically adsorbed onto its surface. Additionally, contact angle (CA) measurements of PDVB revealed a value of approximately 150.10 °, indicating a superhydrophobic character (Figure S6). To further validate the water diffusion enhancement induced by PDVB, we simulated the desorption and diffusion behavior of water in a realistic fixed‐bed environment, using a model dehydration experiment involving CuSO_4_·5H_2_O, which undergoes a distinct color change from blue to white upon losing its crystallization water [25]. The progression of water desorption was monitored by recording the color change of CuSO_4_·5H_2_O over time (Figure 3c‐A). For comparison, the same experiment was conducted under identical conditions without the addition of PDVB (Figures 3c‐B). The results clearly showed that the presence of even a small amount of PDVB significantly accelerated the dehydration process. Collectively, all results presented in Figure 3 indicate that physically mixing PDVB with the inverse catalyst enhances water desorption and diffusion from the catalyst surface, thus minimizing the negative effect of water on catalytic activity.

### 3.4 Time‐on‐Stream Analysis and Characterization of the Spent Catalysts

**FIGURE 4:**
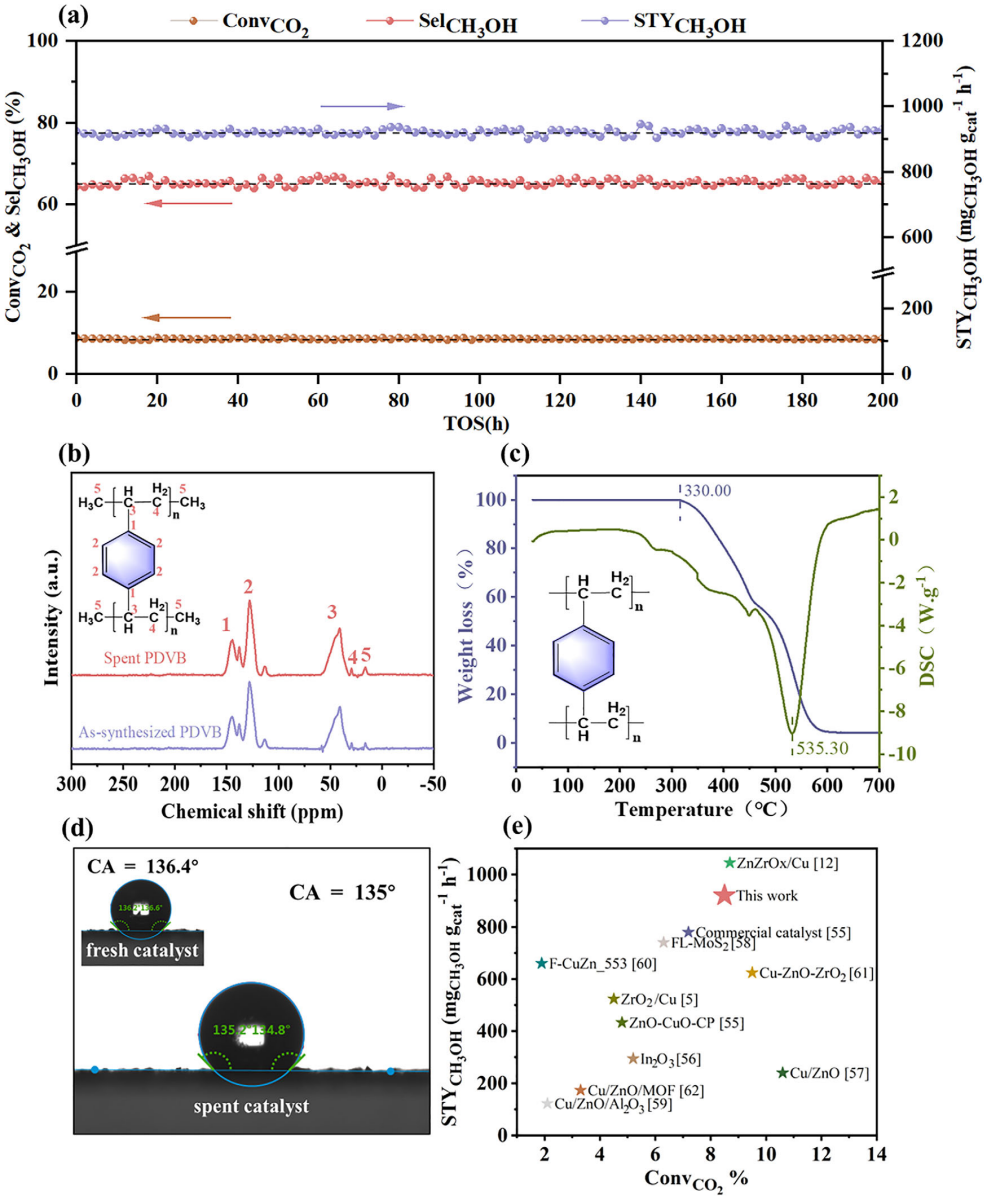
Stability and outstanding performance of Z/C‐P (1:1). (a) The performance of 200 h stability test of Z/C‐P (1:1) catalyst. (b) ^13^C NMR spectra of the as‐synthesized and the spent PDVB components. (c) TGA‐DSC curves of PDVB. (d) Water droplet contact angle (CA) comparison of fresh and 200‐h tested Z/C‐P (1:1) catalysts. (e) Catalytic performance comparison of various catalysts from the literature under conditions similar to 240°C, 5 MPa, and 48 000 mL g_cat_^−^^1^ h^−^^1^.

To evaluate the stability of the Z/C‐P (1:1) catalyst, a 200 h test was conducted under typical reaction conditions. As shown in Figure 4a, the catalytic performance remained stable throughout the duration of the test, indicating that the Z/C–P (1:1) catalyst possessed good thermal stability. Given that PDVB might undergo thermal degradation or melting at elevated temperatures, the ^13^C NMR (Figure 4b) and TG‐DSC results (Figure 4c) reveal that PDVB retained its chemical integrity throughout the reaction process and began to exhibit noticeable weight loss only above 330°C. Additionally, a stability test was conducted on PDVB at 240°C for 3 h. The TG results (Figure S10) indicated that PDVB remains stable under reaction temperature. To further confirm the chemical stability of PDVB during the reaction, FT‐IR analyses of both the synthesized and post‐reaction PDVB (Figure S11) demonstrated that PDVB maintains its chemical integrity. These combined results indicate that PDVB remains thermally stable at the reaction temperature of 240°C [25, 54]. The XRD patterns of the reduced and spent catalysts are shown in Figure S12. Metallic copper diffraction peaks are clearly observed in the spent catalyst. Furthermore, the Cu crystallite sizes were approximately 10.81 nm and 11.20 nm for the fresh and spent catalysts, indicating that no significant Cu particle agglomerated [2]. In addition, the contact angle of the spent Z/C‐P(1:1) catalyst was 135.00 °, closely matching the fresh catalyst of 136.40 °, which confirmed that the hydrophobic surface modification remains stable after 200 h. Additionally, we evaluated the Z/C catalyst for up to 200 h under identical conditions (Figure S13); it likewise remained stable, consistent with previous reports [20, 55]. Considering that the performance of both catalysts would not show a noticeable decline within a short period, an external water feed (10 mL min^−^^1^) was introduced to accelerate the comparison. Upon H_2_O addition, the Z/C catalyst exhibited a pronounced loss of activity: the STY_CH3OH_ decreased from 707.27 to about 110.5 mg_CH3OH_ g_cat_^−1^ h^−1^ (Figure S14). In contrast, although the Z/C‐P (1:1) catalyst also declined at high water loadings, the decrease was markedly smaller, indicating that PDVB‐induced hydrophobicity mitigates water‐induced deactivation and preserves more accessible active sites. In summary, these findings demonstrated the structural and functional stability of the Z/C‐P (1:1) catalyst under reaction conditions, supporting its practical applicability for long‐term methanol production. Figure 4e presents a comparative assessment of the methanol productivity and CO_2_ conversion across various catalysts under similar reaction conditions, with corresponding quantitative data detailed in Table S5 [5, 12, 55, 56, 57, 58, 59, 60, 61, 62]. From the statistical results, it can be observed that the performance of this work is comparable to, and in some cases exceeds, that reported in most of the literature. These findings positioned the hydrophobic Z/C‐P (1:1) catalyst as one of the most competitive Cu‐based catalysts for low‐temperature CO_2_‐to‐methanol conversion reported to date.

**FIGURE 5:**
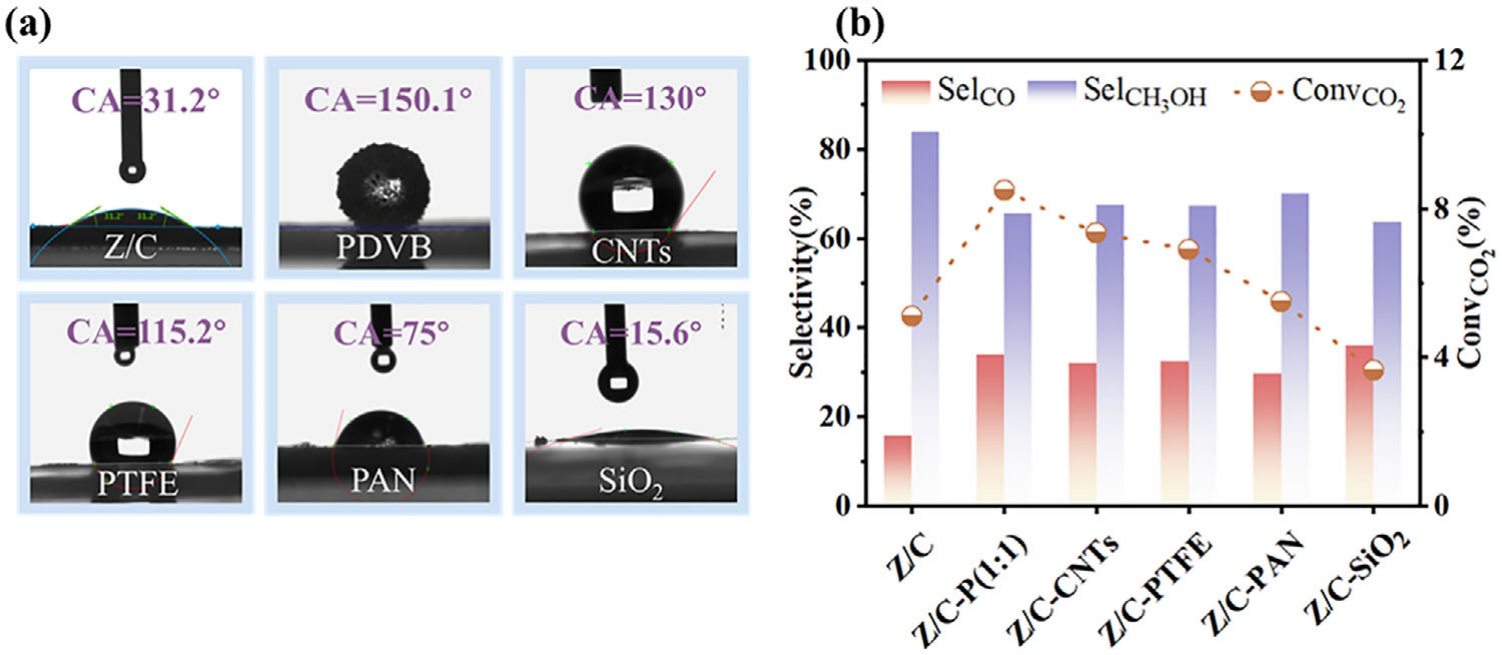
Evaluation of physically mixed Z/C catalyst with inert materials of varying wettability. (a) Water droplet contact angles on various inert materials. (b) Catalytic performance of the Z/C catalyst mixed with various inert materials.

To verify the generality of the hydrophobic modification approach proposed in this study and the specific superiority of PDVB, materials with different wettability were mixed with the Z/C catalyst using the same modification method. These materials included PDVB (water contact angle = 150.1°), CNTs (130.0°), PTFE (115.2°), PAN (75°), and SiO_2_ (15.6°), as shown in Figure 5a. The catalytic performance of these composites was then compared under the same reaction conditions to investigate the correlation between wettability and catalytic activity (Figure 5b). The results demonstrated that hydrophobic materials such as PDVB, PTFE, and CNTs enhanced the CO_2_ conversion rate from 5.11% to 8.5%, 7.35%, and 6.9%, respectively, while the methanol selectivity gradually decreased. Although the water contact angle of PAN was below 90°, it remained significantly higher than that of the pristine Z/C catalyst, resulting in a moderate improvement in catalytic performance for Z/C–PAN. In contrast, when hydrophilic SiO_2_ was used, the CO_2_ conversion rate decreased to 3.65%. These results indicated that PDVB exhibits the best catalytic performance and confirmed the generality of the hydrophobic modification method in this study.

### 3.5 Mechanistic Insights Via In Situ DRIFTS

**FIGURE 6:**
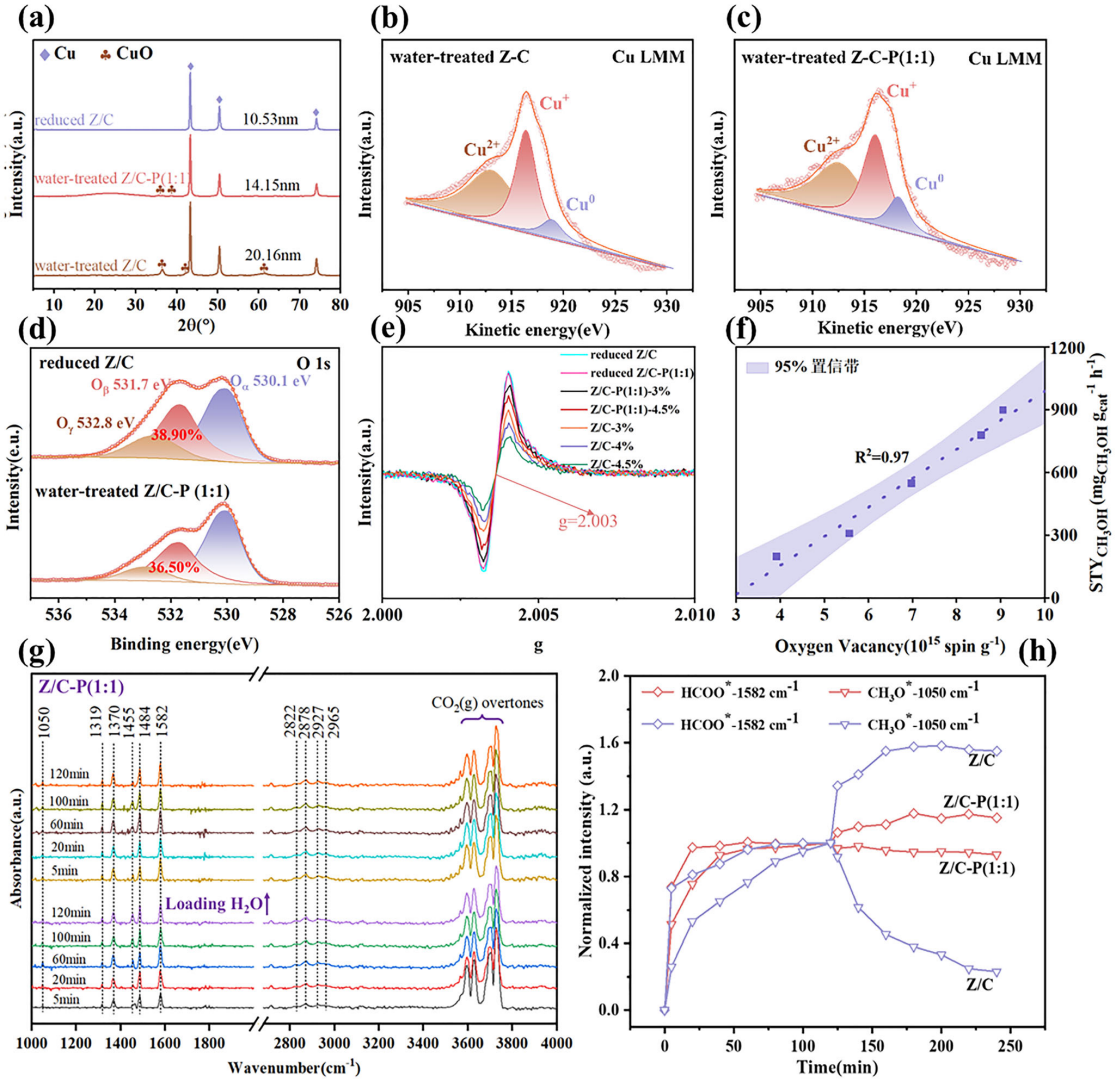
Investigation of the role of water and the reaction mechanism. (a) XRD patterns of reduced Z/C, water‐treated Z/C, and water‐treated Z/C‐P (1:1) catalysts. (b,c) Cu LMM spectra of water‐treated Z/C, and water‐treated Z/C‐P (1:1). (d) O 1s XPS spectra of reduced Z/C and water‐treated Z/C‐P (1:1). (e) EPR signals of the different catalysts. (f) Correlation between the oxygen vacancy concentration and methanol space–time yield (STY). (g) In situ DRIFTS spectra of Z/C‐P (1:1) during CO_2_ hydrogenation at 240 °C with and without water. (h) The trend of the normalized intensities of the key intermediates over time for the Z/C‐P(1:1) and Z/C catalysts. The intensity of the species is normalized to the steady state of each corresponding infrared wavelength band [5].

To exclude the potential influence of PDVB on the intrinsic structure of Z/C catalyst, systematic characterizations were performed on the reduced Z/C and Z/C‐P (1:1) catalysts. The Cu LMM results (Figure S15) showed that both reduced catalysts were dominated by Cu^0^, and the proportions of different Cu species were essentially consistent [48]. Additionally, the EPR results (Figure S16) revealed that the oxygen vacancy content remained basically identical between the two reduced catalysts [63]. To further verify that PDVB exerted no other effects on the Z/C catalysts, the Cu dispersion of reduced catalysts with varying loadings was compared (Figure S17). The results demonstrated that the hydrophobic modification method employed in this study had no significant impact on the catalyst dispersion. Additionally, the following discussion compared the CO_2_‐TPD and O1s XPS of reduced Z/C and Z/C‐P (1:1) catalysts to further exclude possible interactions between PDVB and the catalysts during high‐temperature reduction. Therefore, the improved performance was attributed to the hydrophobic effect, which preserved more accessible active sites in water‐producing reaction environments by preventing water accumulation. To elucidate the impact of water on catalytically active sites, pathways, and intermediates evolved, we utilized in situ DRIFTS, Cu LMM, and EPR to characterize the microstructure, focusing on the particle size and oxidation state of Cu and oxygen vacancies. A number of literature has indicated that the ZrO_x_‐Cu interface was the active site in inverse ZrO_2_/Cu catalysts [5, 30, 64, 65]. To be more specific, an increase in the Cu surface area would lead to a greater number of ZrO_x_‐Cu interfaces [2]. To clearly reveal the effect of water on the catalyst, we introduced 3 vol% deionized water for water treatment under reaction conditions [51, 66]. As shown in Figure 6a, the water‐treated Z/C‐P (1:1) catalyst shows a significantly smaller particle size (14.15 nm) than the water‐treated Z/C catalyst with 20.16 nm, highlighting that the hydrophobic PDVB modification effectively inhibits Cu particle aggregation. This resulted in a higher surface area and thus better catalytic performance. Additionally, the Cu LMM (Figure 6b,c) reveals the water‐treated Z/C catalyst has the highest proportion of Cu^2^^+^, with a nearly complete loss of Cu^0^, indicating the water treatment promotes the oxidation of Cu, diminishing its catalytic activity. In contrast, the Z/C‐P (1:1) catalyst maintained a higher proportion of Cu^0^, suggesting that PDVB modification promoted to maintain Cu in its metallic state [26]. The above results indicated that the Z/C‐P (1:1) catalyst showed excellent surface area and oxidation resistance in the presence of water. Under identical reaction conditions, the STY_CH3OH_ of the Z/C‐P(1:1) catalyst was consistently higher than that of the Z/C catalyst. Additionally, at 5 Mpa and 48,000 mL·g_cat_^−1^·h^−1^, the apparent activation energies of the Z/C‐P(1:1) and Z/C catalysts were determined by varying the reaction temperature (Figure S18). The activation energy of the Z/C‐P(1:1) catalyst was lower than that of the Z/C catalyst, indicating that CO_2_ hydrogenation to methanol occurred more easily on the modified Z/C‐P(1:1) catalyst, which is consistent with the previous findings [26].

Unlike conventional studies that primarily focus on the oxidation of Cu active sites, this work further investigates the impact of hydrophobic modification on oxygen vacancies‐a key factor in inverse catalytic interfaces‐to provide a deeper understanding of the regulation mechanism on inverse interfaces. Oxygen vacancies (O_V_) served as active sites for the adsorption and activation of CO_2_ [20, 67, 68, 69]. The O 1s XPS spectra, as shown in Figure 6d, exhibit peaks at 530.1, 531.7, and 532.8 eV, corresponding to lattice O atoms (O_α_), oxygen vacancies (O_β_), and surface hydroxyl groups ^*^OH (O_γ_), respectively [70, 71]. Peak‐fitting analysis revealed that the oxygen vacancy content of the Z/C catalyst prior to reaction was 38.90%. Following the water treatment, the Z/C‐P (1:1) catalyst exhibited an oxygen vacancy content of 36.50%, which was similar with the reduced Z/C catalyst. However, the oxygen vacancy content in the water‐treated Z/C catalyst decreased to 27.50% (Table S6). Furthermore, the O1s XPS results of the reduced Z/C‐P (1:1) catalyst (Figure S19) were similar to those of the reduced Z/C catalyst, further confirming that PDVB only serves as a hydrophobic modifier for the Z/C catalyst. To further investigate the evolution of surface defects and their impact on catalytic performance, Electron Paramagnetic Resonance (EPR) measurements were performed on the catalysts treated under different water‐treated conditions. Apart from the reduced Z/C‐P(1:1) and reduced Z/C, all other catalysts were pre‐treated for 12 h using various levels of added water, with these values reflected by the percentages specified. As illustrated in Figure 6e, all catalysts exhibited a distinct and symmetric resonance signal at g = 2.003, which is characteristic of unpaired electrons trapped in oxygen vacancies (Ov) [12, 55, 72]. Specifically, the Z/C‐P(1:1) series maintained a substantially higher density of Ov compared to the unmodified Z/C series. Regarding the Z/C catalyst, the intensity of oxygen vacancies (Ov) exhibited a pronounced decline with the increase in added water content from 3% to 4.5%. This indicated that while water led to the consumption or elimination of surface oxygen vacancies, the hydrophobic PDVB effectively protected the active sites from water‐induced degradation. Furthermore, for all water‐treated catalysts, the quantitative Ov concentration was correlated with the methanol space‐time yield. The relationship between oxygen vacancy concentration and catalytic performance is shown in Figure 6f, demonstrating a direct correlation. This indicated that oxygen vacancies act as the primary active sites for CO_2_ hydrogenation to methanol, and the PDVB modification could effectively preserve oxygen vacancies. This hydrophobic effect prevented water from blocking the oxygen vacancies, thereby maintaining the availability of active sites crucial for CO_2_ activation. Furthermore, CO_2_‐TPD analysis was performed on the reduced catalysts and the Z/C‐P (1:1) and Z/C catalysts treated with 3% added water (Figure S20). The number of basic sites remained almost the same between the reduced Z/C and Z/C‐P (1:1) catalysts, indicating that the intrinsic basicity of the Z/C catalyst was not altered by the addition of PDVB. And the results showed that, under the same water treatment conditions, the number of weak and medium basic sites of the modified Z/C‐P(1:1) catalyst (0.298 mmol/g) is significantly higher than that of the Z/C catalyst (0.176 mmol/g), and is almost identical to that of the catalyst before reaction (0.322 mmol/g). These results further confirmed that PDVB effectively suppresses the negative impact of water on CO_2_ activation, leading to an increased CO_2_ conversion rate for the modified catalyst.

Currently, two main pathways of metal oxide‐based catalysts for CO_2_ hydrogenation to methanol have been widely accepted: the formate (HCOO^*^) pathway and the CO‐mediated reverse water‐gas shift (CO–RWGS) pathway [73, 74, 75]. Combined with the aforementioned evidence that PDVB did not alter the intrinsic properties of the Z/C catalyst, it was theoretically expected that Z/C‐P (1:1) catalyst would follow the same reaction pathway as Z/C. To investigate the reaction mechanism and the impact of water on the evolution of reaction intermediates, in situ DRIFTS was conducted at 240 °C under both with and without water conditions. In our study, both formate (HCOO^*^) and methoxy (CH_3_O^*^) intermediates were observed over Z/C and Z/C–P (1:1) catalysts, while almost no discernible IR signals attributable to adsorbed COOH^*^ and CO^*^ species were detected. This suggested that methanol formation over both catalysts predominantly would follow the formate pathway, which is consistent with previous studies [5, 18, 30, 34, 76]. The mechanism involved the stepwise hydrogenation and dehydration of CO_2_^*^‐derived intermediates (CO_3_^*^, HCOO^*^, CH_3_O^*^) to produce methanol.

To clearly investigate the mechanistic influence of water on the evolution of surface reaction intermediates, 3 vol% H_2_O was introduced into the reaction system. The results are as shown in Figure 6g and Figures S21 and S22. The characteristic peaks corresponding to different surface intermediates are summarized in Table S7. The DRIFTS spectra for the hydrophobically modified Z/C–P (1:1) catalyst (Figure 6g) exhibited negligible spectral changes upon water introduction. While a slight increase in HCOO^*^ bands (1319, 1370, 1582, 2878, 2965 cm^−1^) was observed, the intensity of CH_3_O^*^ bands (1050, 2822, 2927 cm^−1^) remained essentially unchanged [77, 78, 79, 80, 81, 82, 83, 84]. However, on the Z/C catalyst, the introduction of water vapor led to a significant increase in the intensity of bands corresponding to HCOO^*^ species and CO_3_^*^ species (1455 cm^−1^) [64]. In contrast, the methoxy (CH_3_O^*^) signals (1050, 2822, 2927 cm^−1^) were initially weak and decreased markedly upon the introduction of external water [80, 83, 84]. This observation was consistent with previous reports that water inhibited the formate intermediates hydrogenation to methoxy species, the key precursor to methanol [22, 77]. As the rate‐limiting step, formate hydrogenation decided the overall efficiency of the interfacial catalytic process [76]. The accumulation of HCOO^*^ species on the catalyst surface may block active sites, thereby reducing methanol productivity. To futher provided molecular‐level insights into the hydrophobic modification mechanism, the evolution of surface species in the 3000–3900cm^−1^ region (‐OH stretching vibrations) was monitored. As shown in Figure 6g and Figure S22, for the unmodified Z/C catalyst, the broad band emerged at 3000–3570cm^−1^, attributed to the co‐contribution of physically adsorbed water and hydrogen‐bonded associated hydroxyl groups, indicating the formation of an over‐saturated hydration layer [85, 86, 87]. These dense water species competitively occupied the oxygen vacancies of the interface and created a physical barrier that inhibits hydrogen spillover and the subsequent hydrogenation of formates to methoxy species. In contrast, the Z/C–P (1:1) catalyst exhibited no significant broad band in this region, with the intensity of gas‐phase H_2_O structures at 3522cm^−1^ and 3525cm^−1^ remaining nearly identical to the pre‐water introduction stage [86, 88]. This directly demonstrated that the PDVB effectively facilitated the desorption and diffusion of water molecules, maintaining an active interface and inhibits the competitive adsorption and site‐blocking effects of external water molecules.

Additionally, we tracked the intensity of key intermediates over time, which can provide time‐dependent structural evolution of these intermediates to clarify the mechanistic implications. As shown in Figure 6h, upon rapid exposure to the reaction atmosphere, the typical HCOO^*^ and CH_3_O^*^ intermediates on the Z/C‐P(1:1) catalyst quickly reached steady state. In contrast, the same intermediates on the unmodified Z/C catalyst took a longer time to reach steady state, further indicating that the Z/C‐P(1:1) catalyst accelerates the hydrogenation conversion of intermediates. After water was introduced, the changes in the HCOO^*^ and CH_3_O^*^ species on the Z/C‐P(1:1) catalyst were minimal compared to the steady‐state values without water. On the unmodified Z/C catalyst, however, the intensity of HCOO^*^ increased nearly 1.6 times, and CH_3_O^*^ clearly decreased. This phenomenon further demonstrates that the hydrophobic PDVB modification effectively protects the hydrogenation step of HCOO^*^ to CH_3_O^*^. The in situ DRIFTS results provided direct microscopic evidence that the PDVB modification effectively eliminates water‐induced inhibition of the HCOO^*^ to CH_3_O^*^ conversion step, thereby facilitating continuous methanol formation.

**FIGURE 7:**
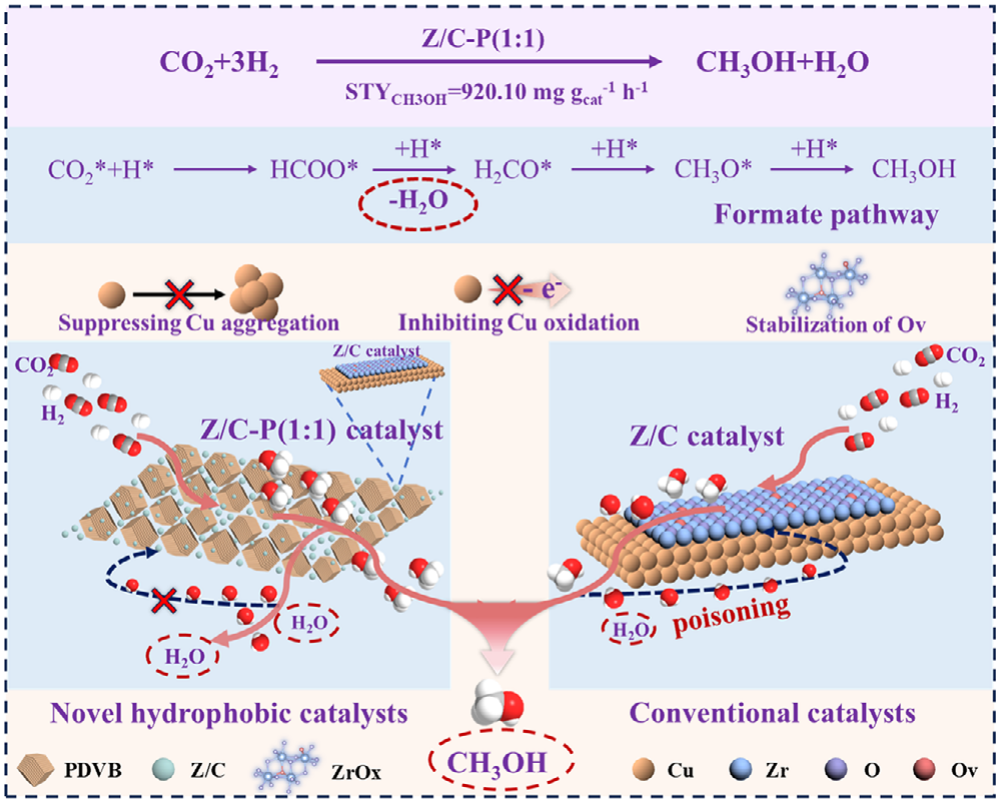
Schematic illustration of the hydrophobic properties and catalytic mechanism of the Z/C–P (1:1) catalyst.

The mechanistic model is shown in Figure 7, in the formate reaction pathway, the water predominantly generated during the transformation of HCOO^*^ to H_2_CO^*^ led to the deactivation of the hydrophilic Z/C catalyst due to the accumulation. To address this issue, we innovatively introduced a physical mixture of hydrophobic polymer PDVB with the hydrophilic Z/C catalyst, which effectively promoted water desorption and rapid diffusion. This local dehydration on the catalyst surface significantly enhanced the catalytic performance, achieving a high methanol space‐time yield of 920.10 mg_CH3OH_ g_cat_^−^^1^ h^−^^1^. Based on in situ DRIFTS and other characterization techniques, it could be concluded that the incorporation of PDVB not only mitigated the inhibitory effects of water on the rate‐determining hydrogenation of HCOO^*^ but also preserved the particle sizes and metallic state of Cu and the abundance of oxygen vacancy by limiting the interaction with water molecules, thus maintaining the activity of the catalysts.

## 4 Conclusions

Inverse catalysts have attracted growing interest in CO_2_ hydrogenation. However, their hydrophilic surfaces hinder the catalytic performance, and systematic investigations into their surface wettability remain notably lacking. By contrasting with PDVB‐promoted conventional systems, this work established that hydrophobic regulation provided superior benefits for inverse configurations by specifically modulating the continuous oxide‐metal interface. In this work, a facile and non‐destructive hydrophobic modification strategy was successfully applied to inverse ZrO_2_/Cu catalysts via physical mixing with PDVB, yielding a series of hydrophobic ZrO_2_/Cu‐PDVB materials. Among these, the catalyst with a 1:1 mass ratio of ZrO_2_/Cu to PDVB exhibited a remarkable methanol space–time yield (STY) of 920.10 mg_CH3OH_ g_cat_^−^^1^ h^−^^1^ under 240 °C, 5 MPa, and 48 000 mL g_cat_^−^^1^ h^−^^1^, outperforming an approximate 30% enhancement over the unmodified ZrO_2_/Cu catalyst (707.27 mg_CH3OH_ g_cat_^−^^1^ h^−^^1^). This outstanding performance positions it among the most efficient Cu‐based catalysts reported for CO_2_ hydrogenation to methanol.

Mechanistic investigations, including in situ DRIFTS, revealed that the PDVB additive effectively promoted the desorption and outward diffusion of water produced during the reaction. This local dehydration effect not only alleviated the negative effect of water on the rate‐determining step of the formate hydrogenation, but also maintained the size and metallic state of the Cu particles as well as the abundance of oxygen vacancies, which were critical for preserving the active ZrO_x_‐Cu interface. Structure‐activity relationship further revealed that the spatial proximity between PDVB domains and catalytically active sites was essential for achieving optimal performance, suggesting the importance of local microenvironmental control in catalyst design. The optimized catalyst also demonstrated outstanding 200 h thermal stability. Overall, we established the novelty of PDVB‐assisted hydrophobic engineering for inverse catalysts and attributed the about 30% gain to interfacial microenvironment regulation. This approach provides new insights into effective catalyst design, particularly for reactions that are thermodynamically or kinetically inhibited by water accumulation.

## Conflicts of Interest

The authors declare no conflicts of interest.

## Supporting information




**Supporting File**: advs74433‐sup‐0001‐SuppMat.docx

## Data Availability

The data that support the findings of this study are available from the corresponding author upon reasonable request.
