# Supplementary material for: Non‐Destructive Hydrophobic Engineering of Inverse Catalysts for Methanol Synthesis from CO2
Source: Adv Sci (Weinh). 2026 Feb 15;13(24):e23915. doi: 10.1002/advs.202523915 (PMC13116108; doi:10.1002/advs.202523915)
Supplement: Supplementary file 1 — Supporting File: advs74433‐sup‐0001‐SuppMat.docx [file ADVS-13-e23915-s001.docx]

**Supporting Information**


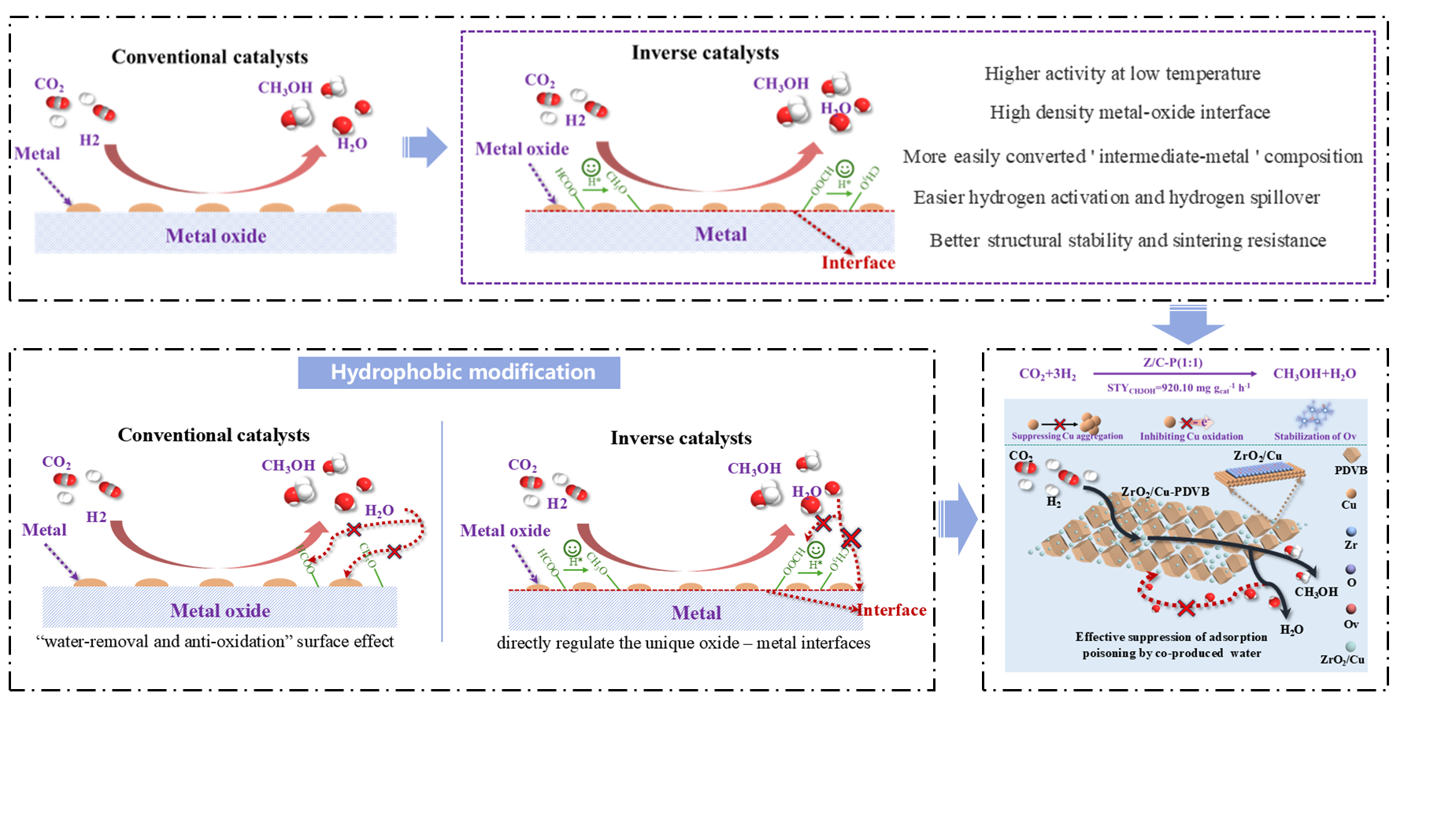


**Figure S1.** Comparison of hydrophobic modification on inverse ZrO_2_/Cu catalysts (this work) versus conventional catalysts in previous works.


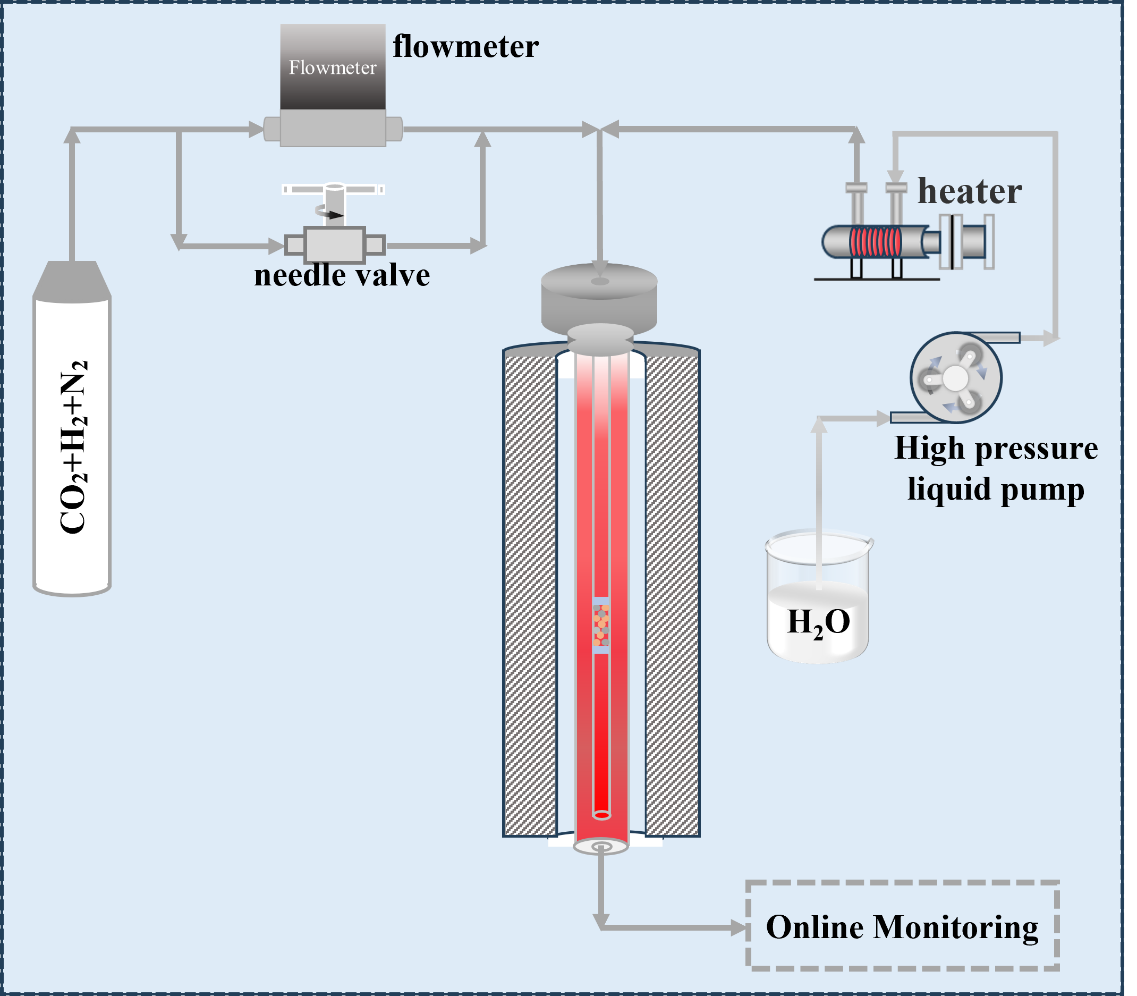


**Figure S2.** Schematic diagram of the fixed-bed reactor system equipped with external water feeding


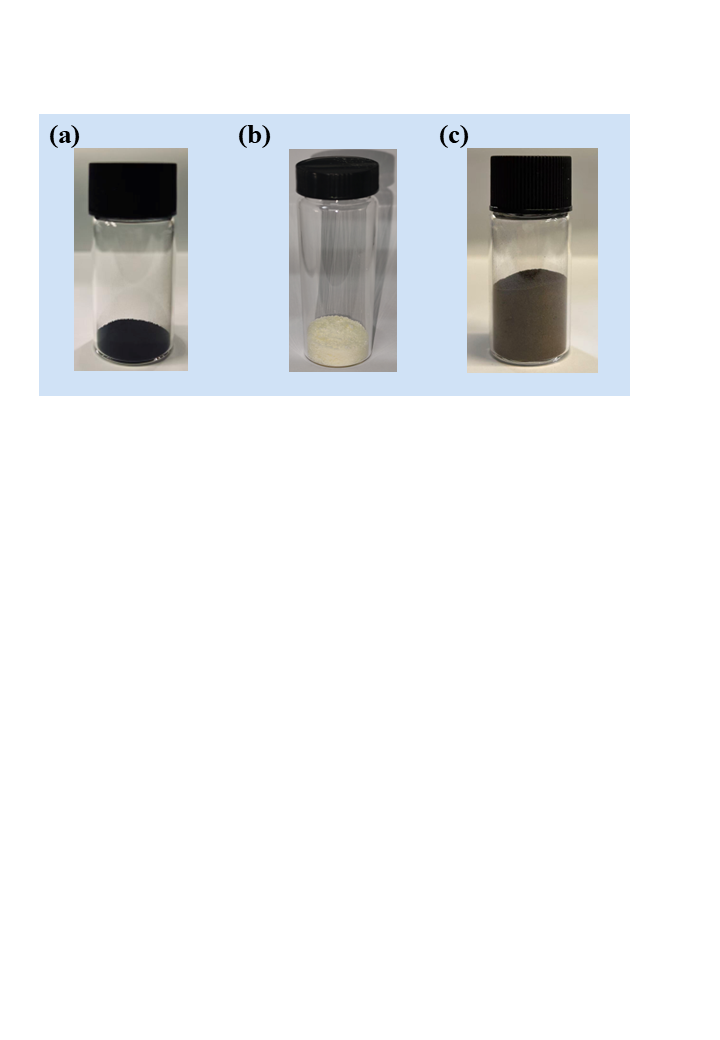


**Figure S3.** The photographs of the catalysts and PDVB. (a) Photograph of ZrO_2_/Cu. (b) Photograph of the synthesized PDVB. (c) Photograph of the mixture of ZrO_2_/Cu and PDVB.


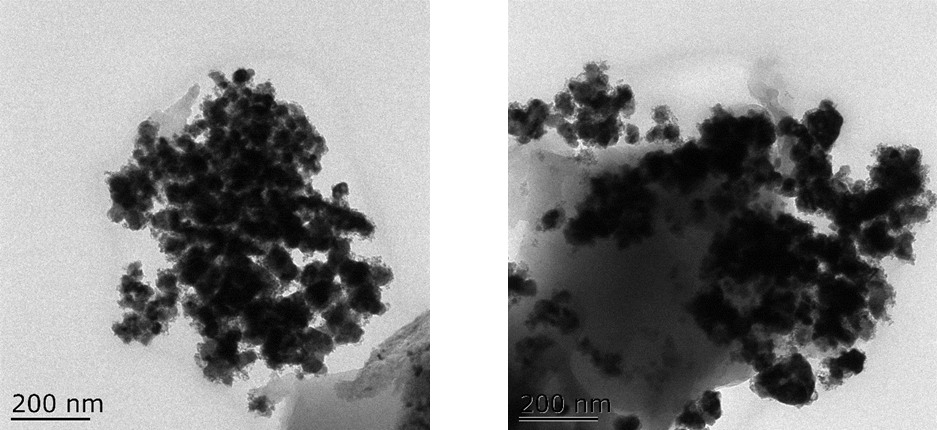


**Figure S4.** The TEM images of Z/C-P (1:1).


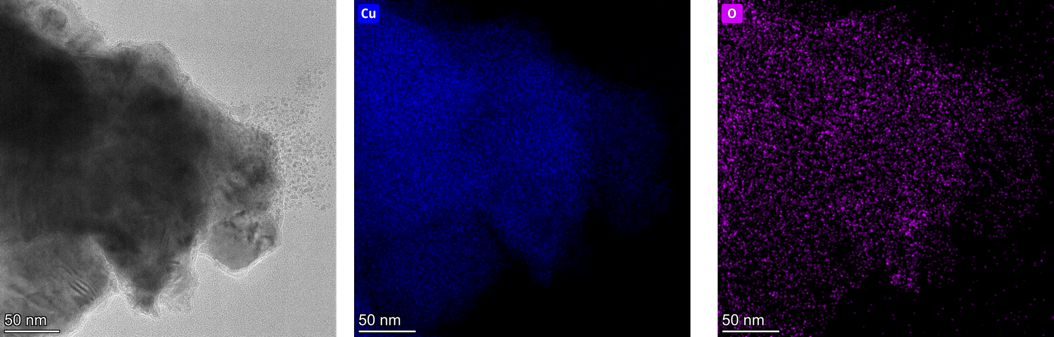


**Figure S5.** The complement of HRTEM + EDS images of the Z/C catalyst.


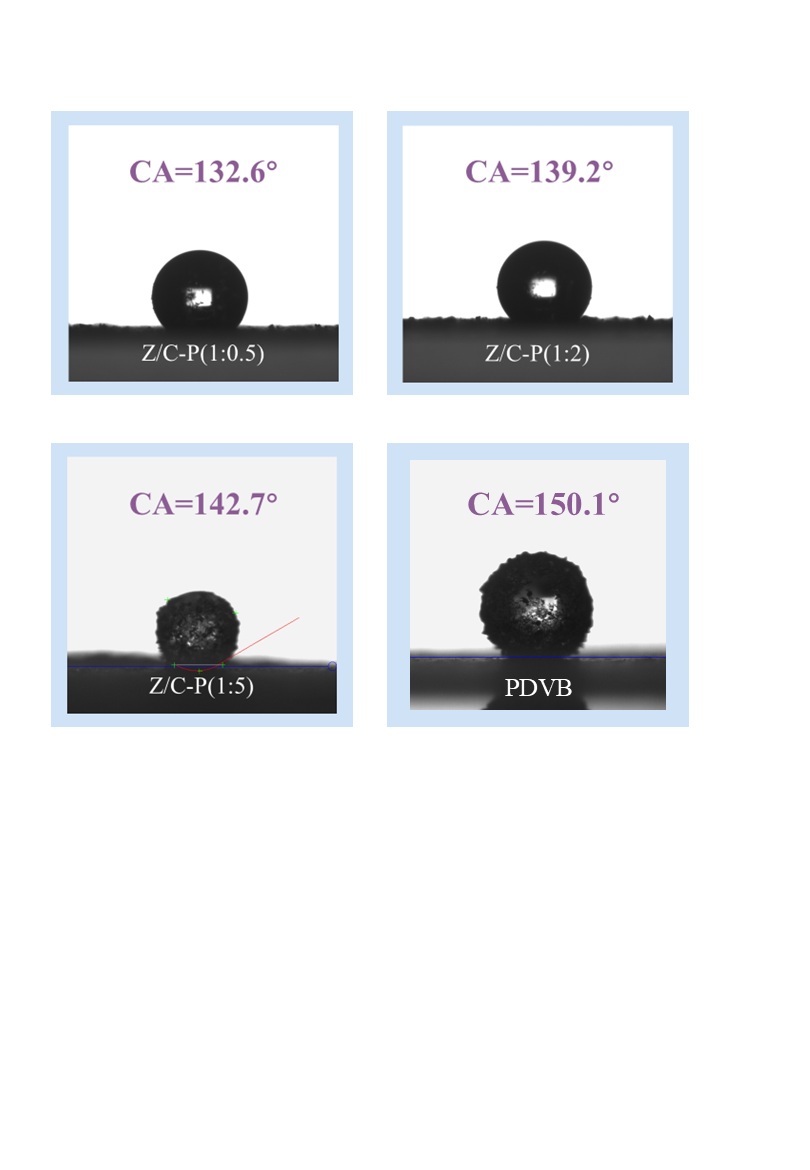


**Figure S6.** The water-droplet contact angles of the Z/C-P (1:0.5), Z/C-P (1:2) and Z/C- P (1: 5).

Note：With the increase of PDVB content, the contact angle of water droplets gradually.


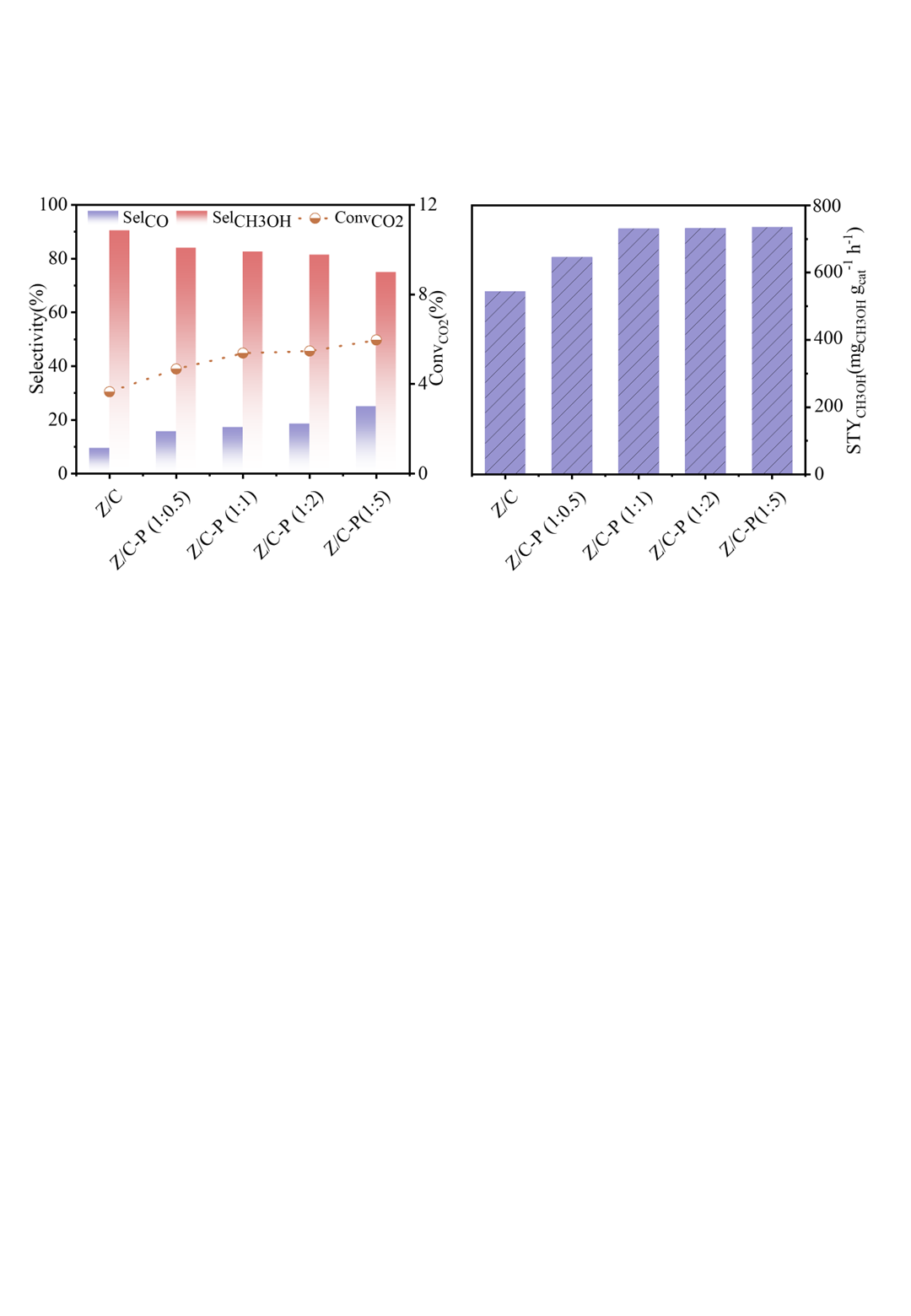


**Figure S7.** Catalytic performance of different catalysts at 220 ℃, 48000 mL g_cat_^-1^ h^-1^, 5 MPa.


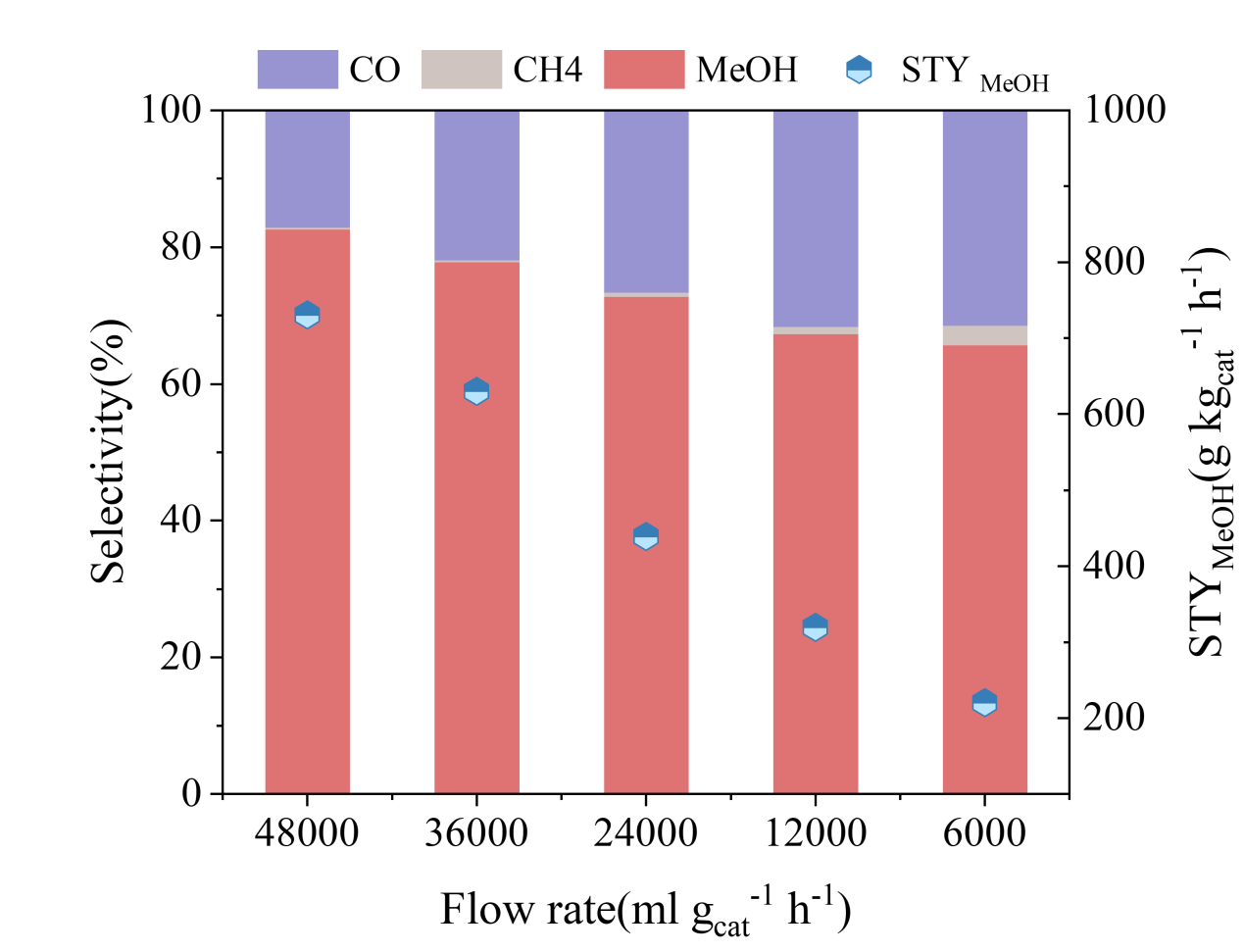


**Figure S8.** The catalytic performance of Z/C-P (1:1) catalyst at 220 ℃, 5 MPa and different GHSV.


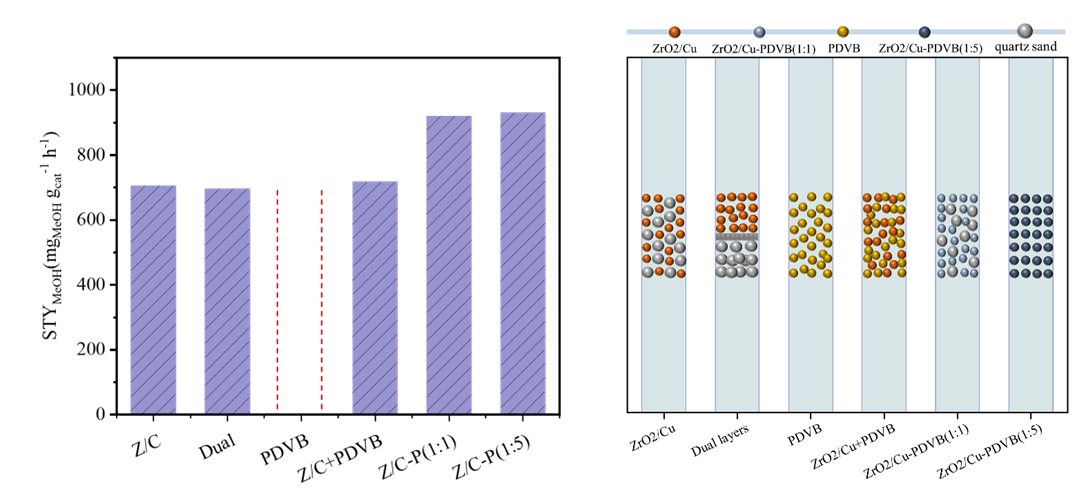


**Figure S9.** STY_CH3OH_ data of different mixing methods.


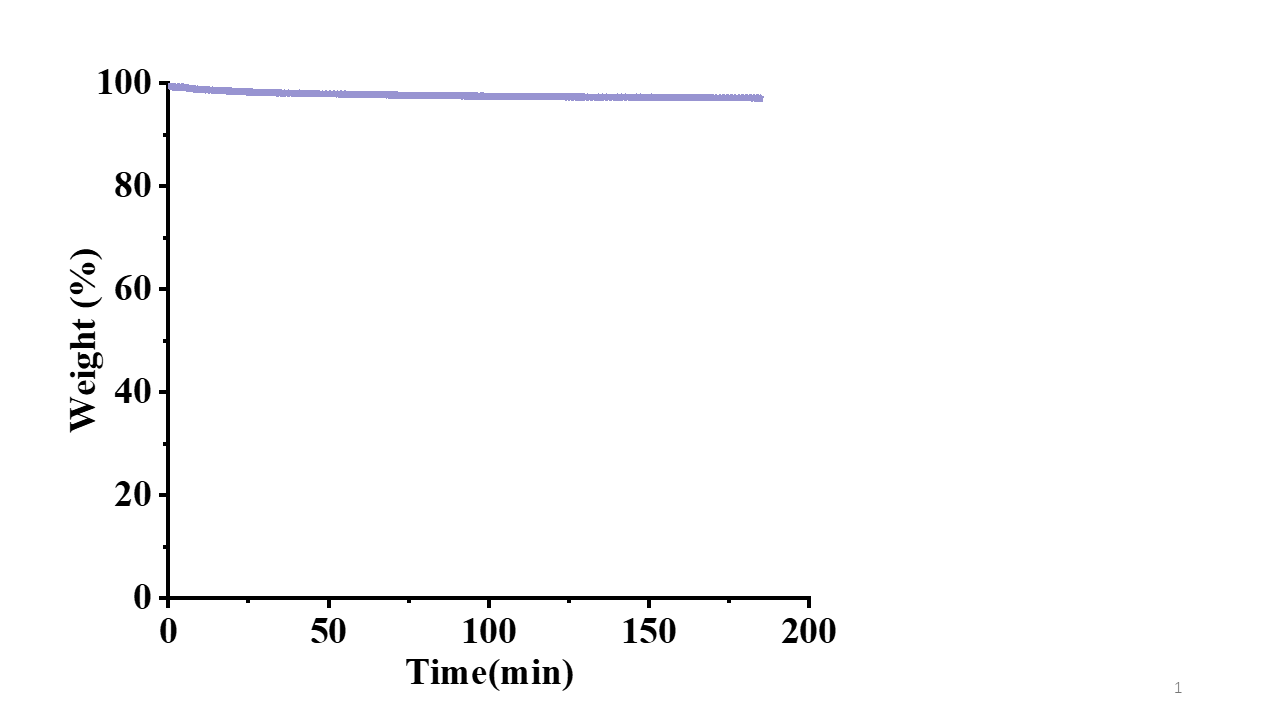


**Figure S10.** Thermogravimetric (TG) results for PDVB at 240 °C over 3 h.


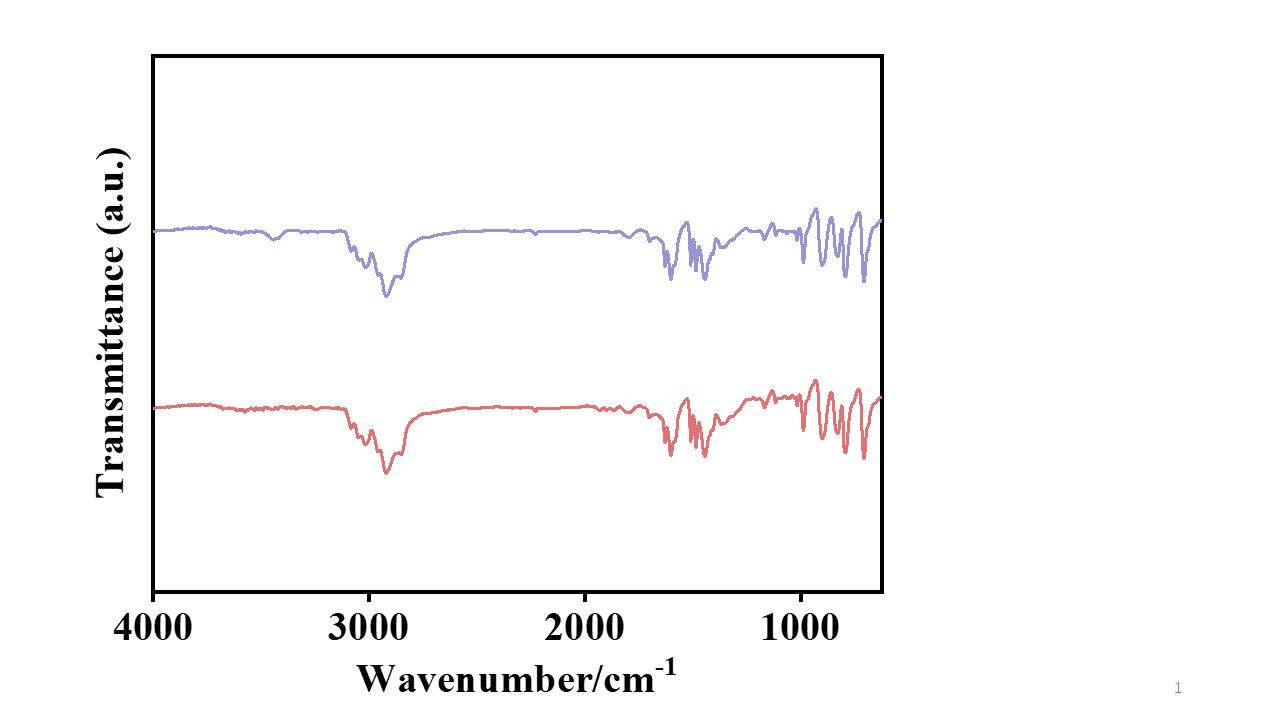


**Figure S11.** FTIR spectra of the as-synthesized and the spent PDVB components


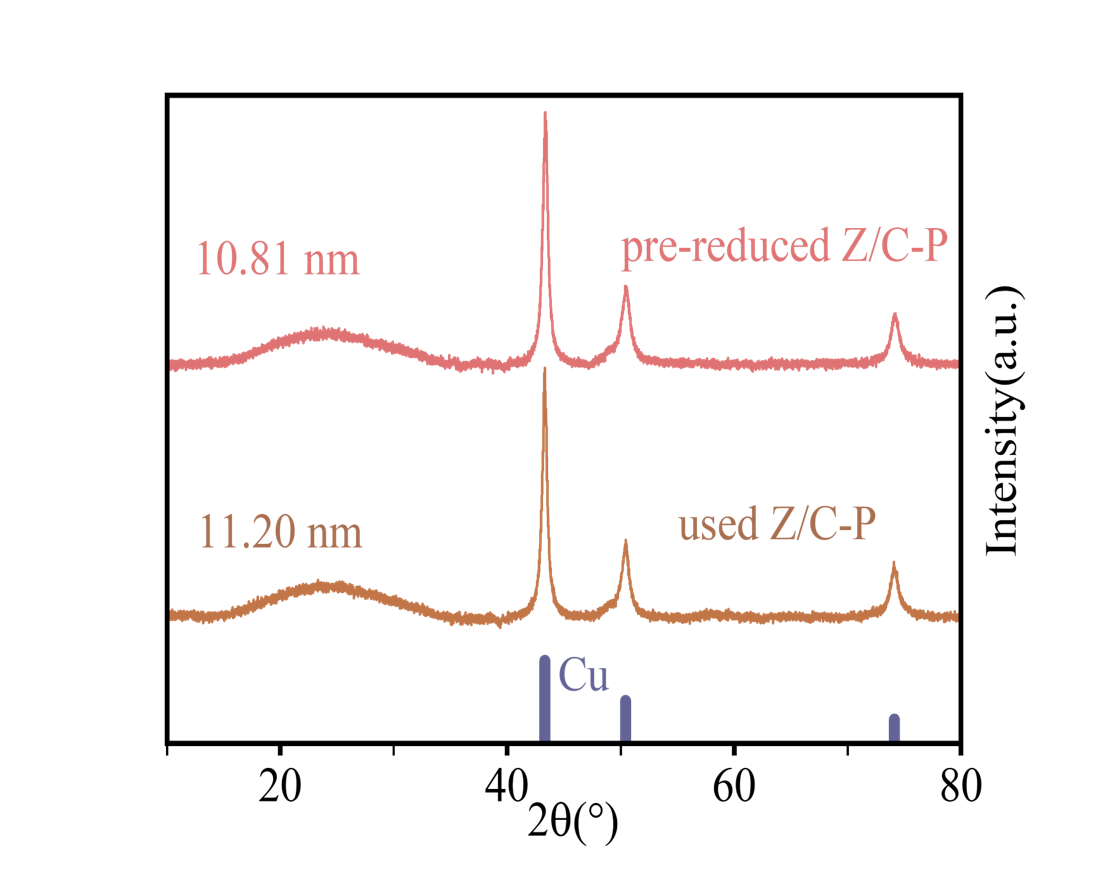


**Figure S12.** XRD comparison of fresh and 200-hour tested Z/C-P (1:1) catalysts.


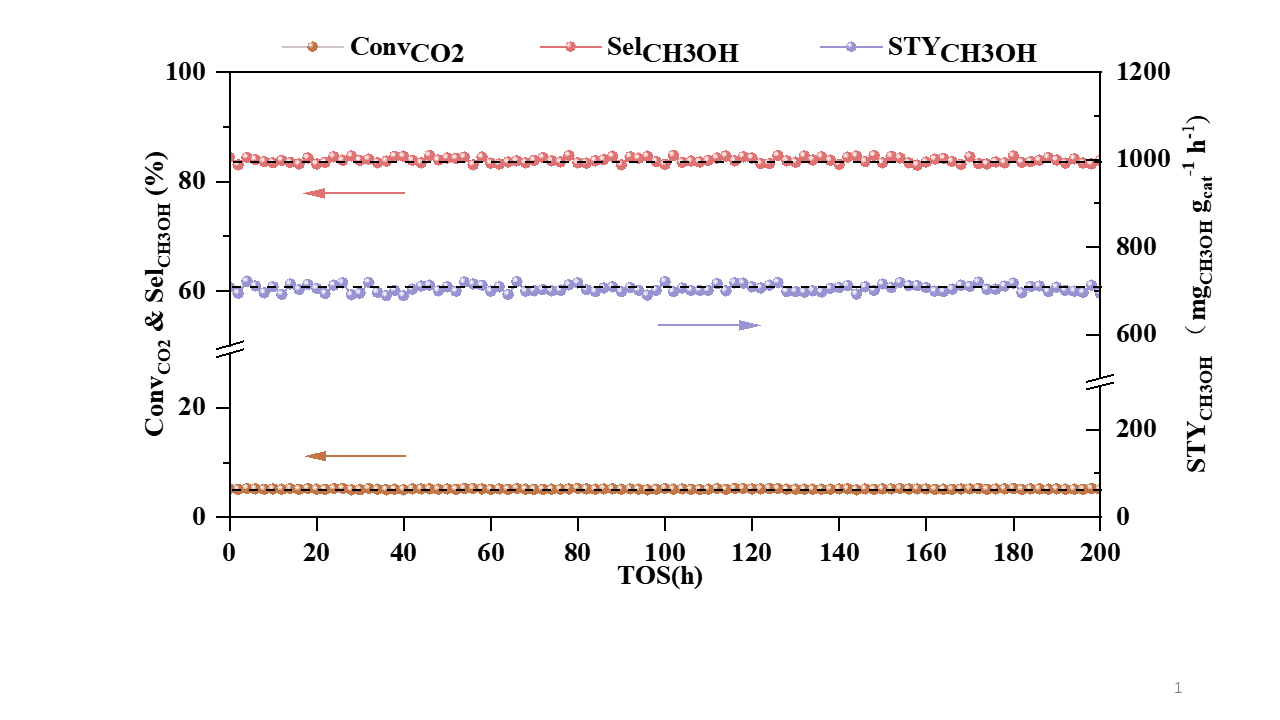


**Figure S13.** The performance of 200 h stability test of Z/C catalyst.


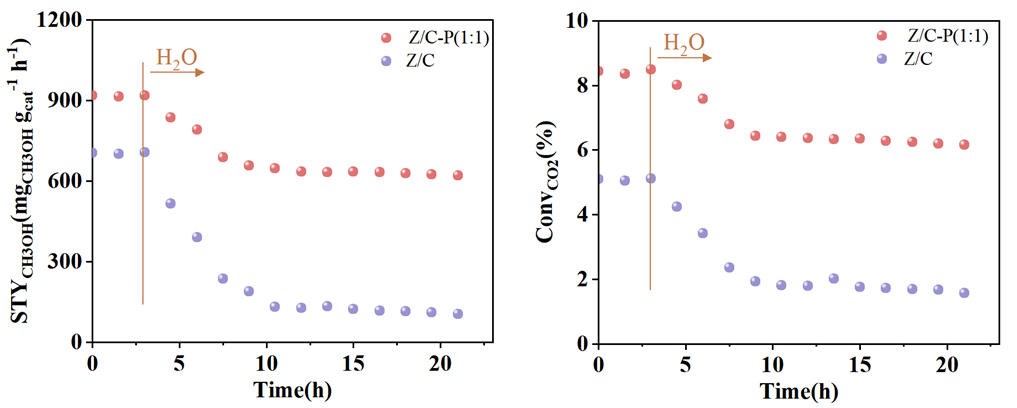


**Figure S14.** Catalytic performance of Z/C and Z/C-P (1:1) upon addition of 10mL/min water. (a) STY_CH3OH_. (b) Conv_CO2_.


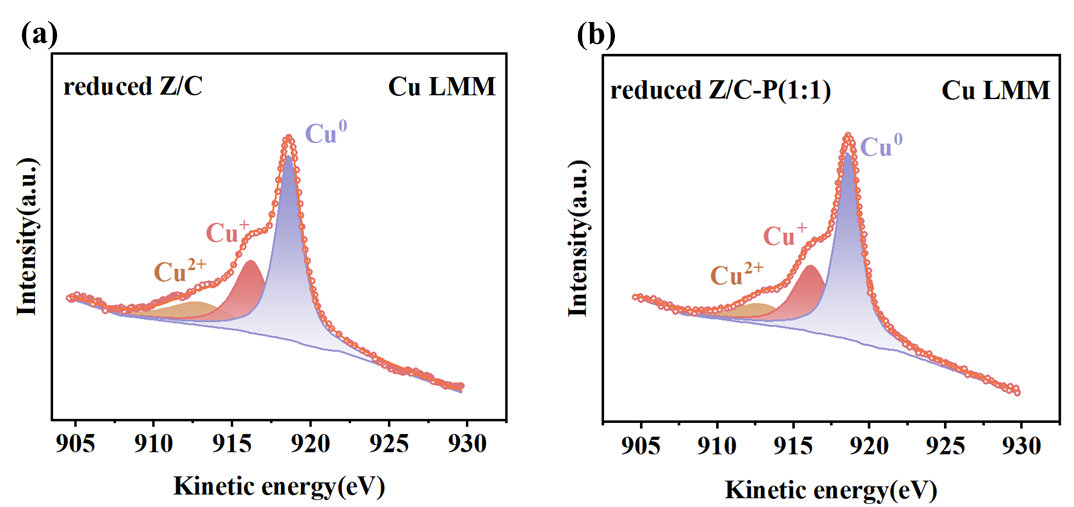


**Figure S15.** Comparison of Cu LMM spectra of the reduced catalysts. (a) Cu LMM spectra of reduced Z/C. (b) Cu LMM spectra of reduced Z/C-P (1:1).


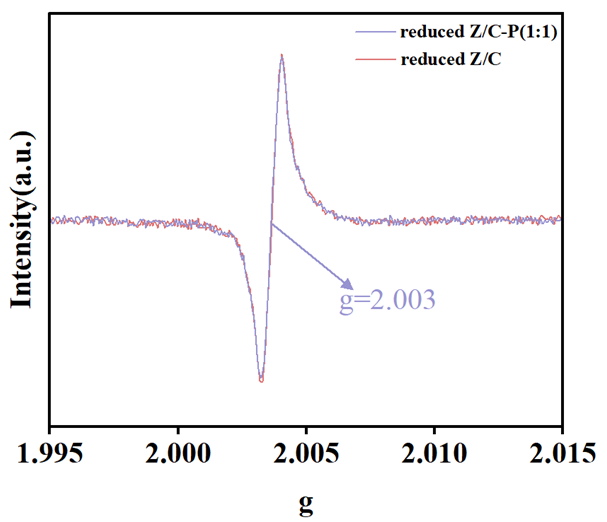


**Figure S16.** EPR signals of the reduced Z/C-P (1:1) and Z/C.


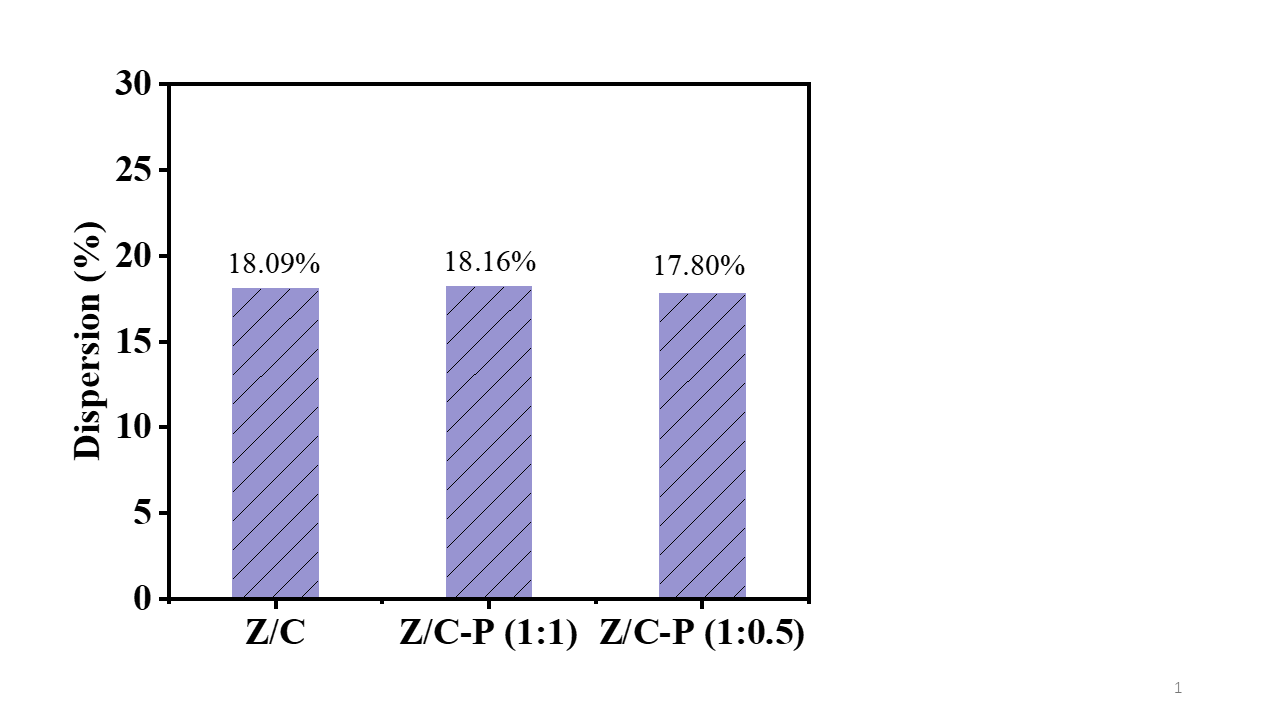


**Figure S17.** Cu Dispersion on the different Catalysts


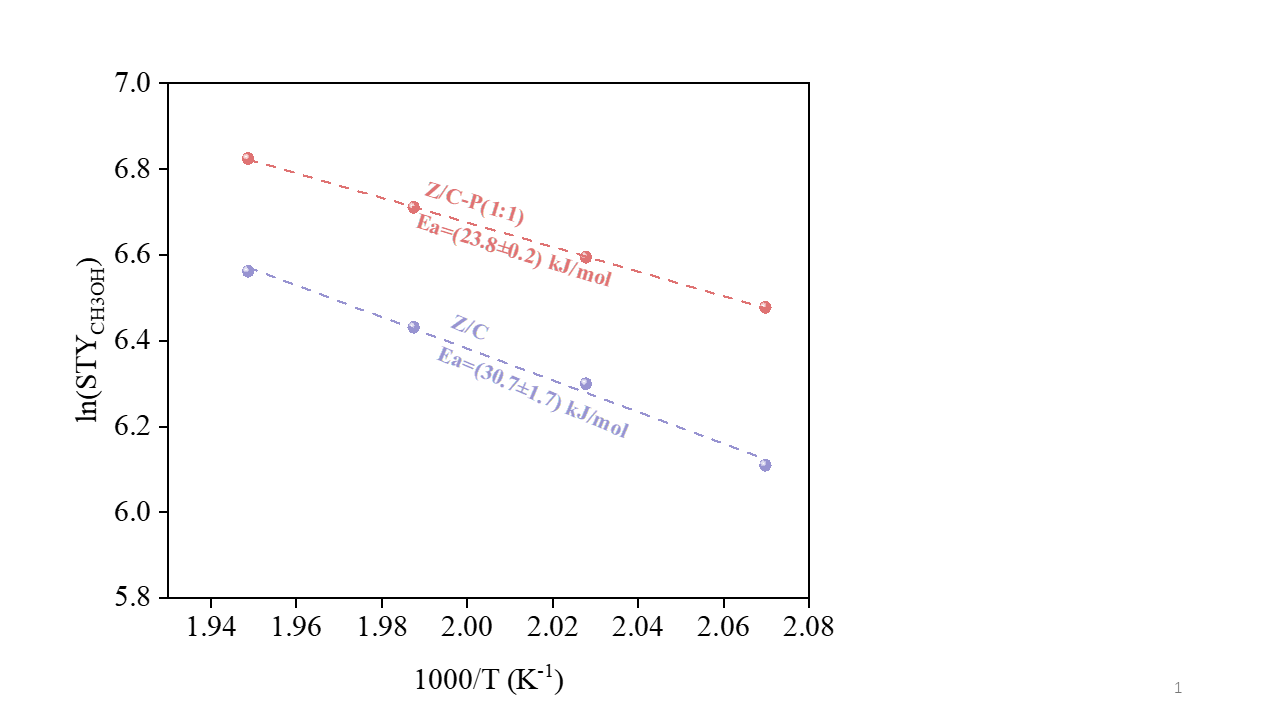


**Figure S18.** Experimental Arrhenius plots for the Z/C-P (1:1) and Z/C catalyst.


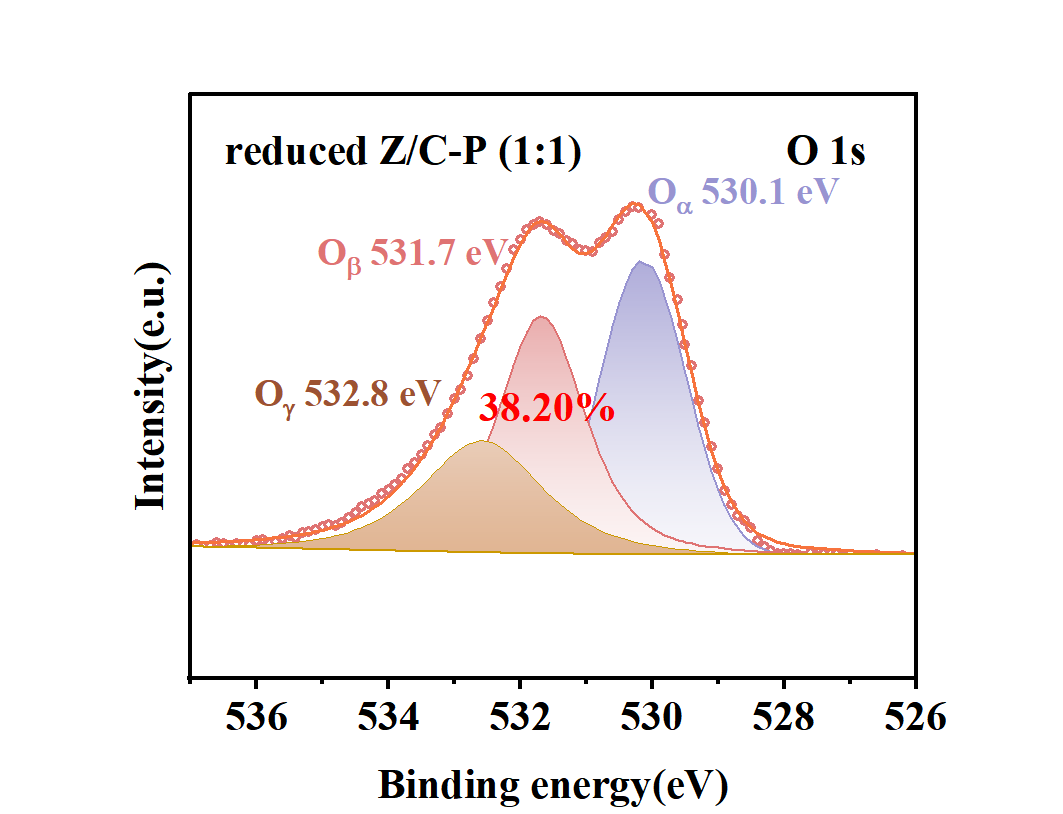


**Figure S19.** O 1s XPS spectra of reduced Z/C-P (1:1)

| 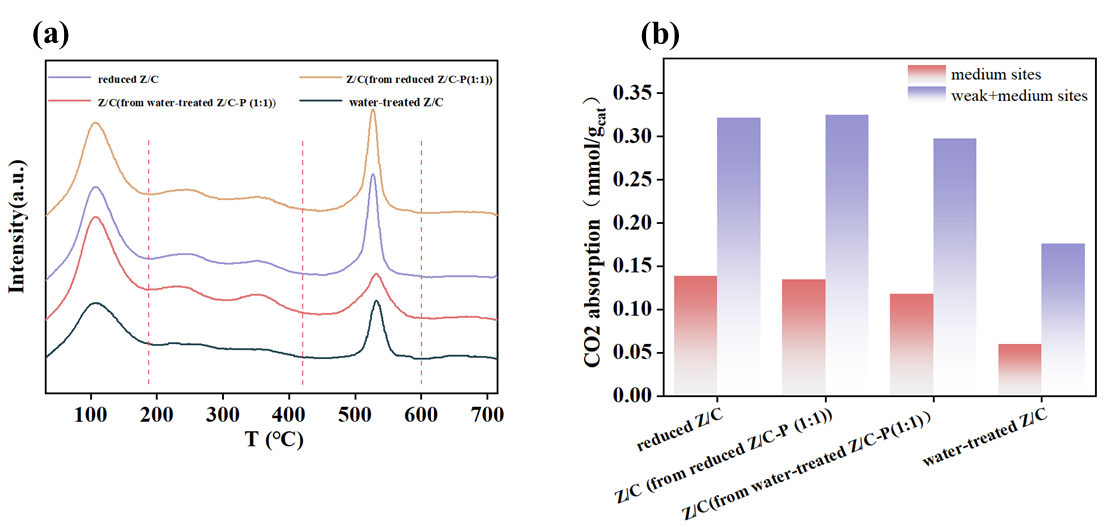 |  |
| --- | --- |

**Figure S20.** The CO_2_-TPD profiles in the reduced catalysts and the water-treated Z/C-P(1:1) and Z/C catalysts. (a) the CO_2_-TPD profiles in different catalysts. (b) the amount of weak and medium adsorbed CO_2_ estimated from the CO_2_-TPD profiles. Note: Owing to the thermal instability of PDVB at elevated temperatures, measurements for the Z/C-P (1:1) catalyst were performed on the separated Z/C instead

To further investigate the effect of the modified Z/C-P(1:1) catalyst on catalytic performance, CO_2_ temperature-programmed desorption (CO_2_-TPD) experiments were conducted on Z/C-P(1:1) and Z/C catalysts subjected to the water treatment. The results are shown in **Fig. S20a**. The CO_2_-TPD profiles can be divided into three desorption peaks, corresponding to the desorption of CO_2_ from weak basic sites (approximately 80–200 °C), medium basic sites (200–400 °C), and strong basic sites (400–640 °C) ^[1, 2]^. Previous studies have shown that the number of weak and medium basic sites is directly related to the catalytic performance ^[1]^. As shown in the **Fig. S20b**, under the same water treatment conditions, the number of weak and medium basic sites of the modified Z/C-P(1:1) catalyst (0.298 mmol/g) is significantly higher than that of the Z/C catalyst (0.176 mmol/g), and is almost identical to that of the catalyst before reaction (0.322 mmol/g). In addition, the CO_2_ adsorption-desorption temperatures did not change significantly, indicating that the hydrophobic modification in this study did not alter the chemical behavior of individual adsorption sites, but rather preserved a greater number of available basic adsorption sites. These results demonstrate that the modified Z/C-P(1:1) catalyst possesses more accessible basic adsorption sites, thereby exhibiting higher CO_2_ conversion efficiency.


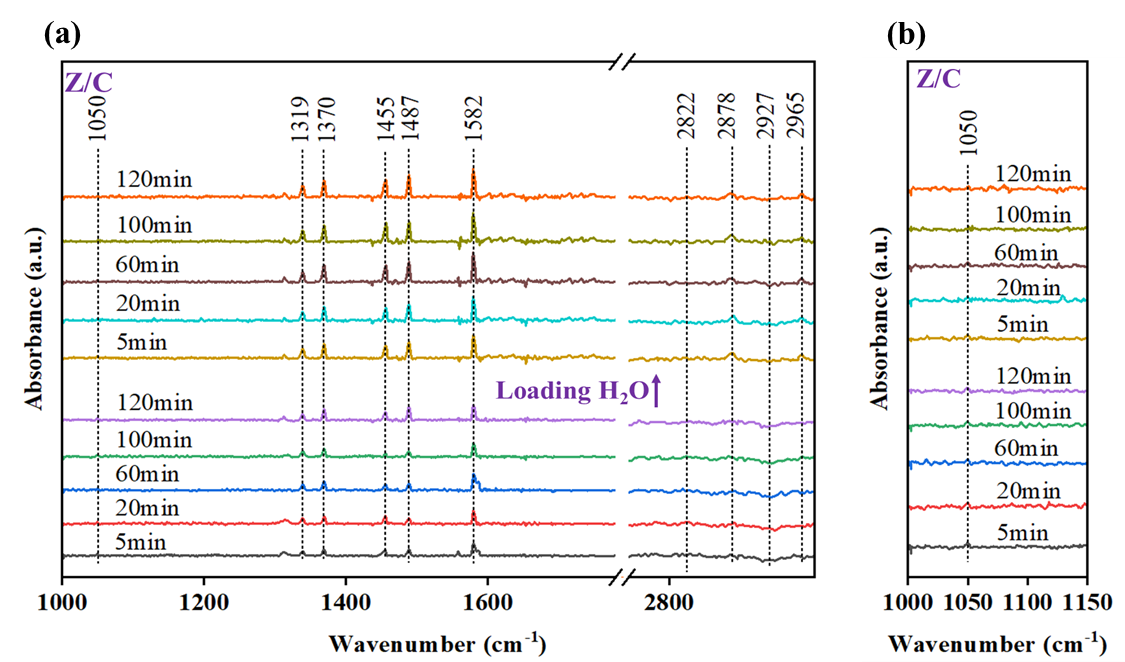


**Figure S21.** (a) In situ DRIFTS spectra of Z/C catalyst during CO_2_ hydrogenation at 240 °C with and without water. (b) Local enlargement of the CH_3_O* vibrational band at 1050 cm^-1^


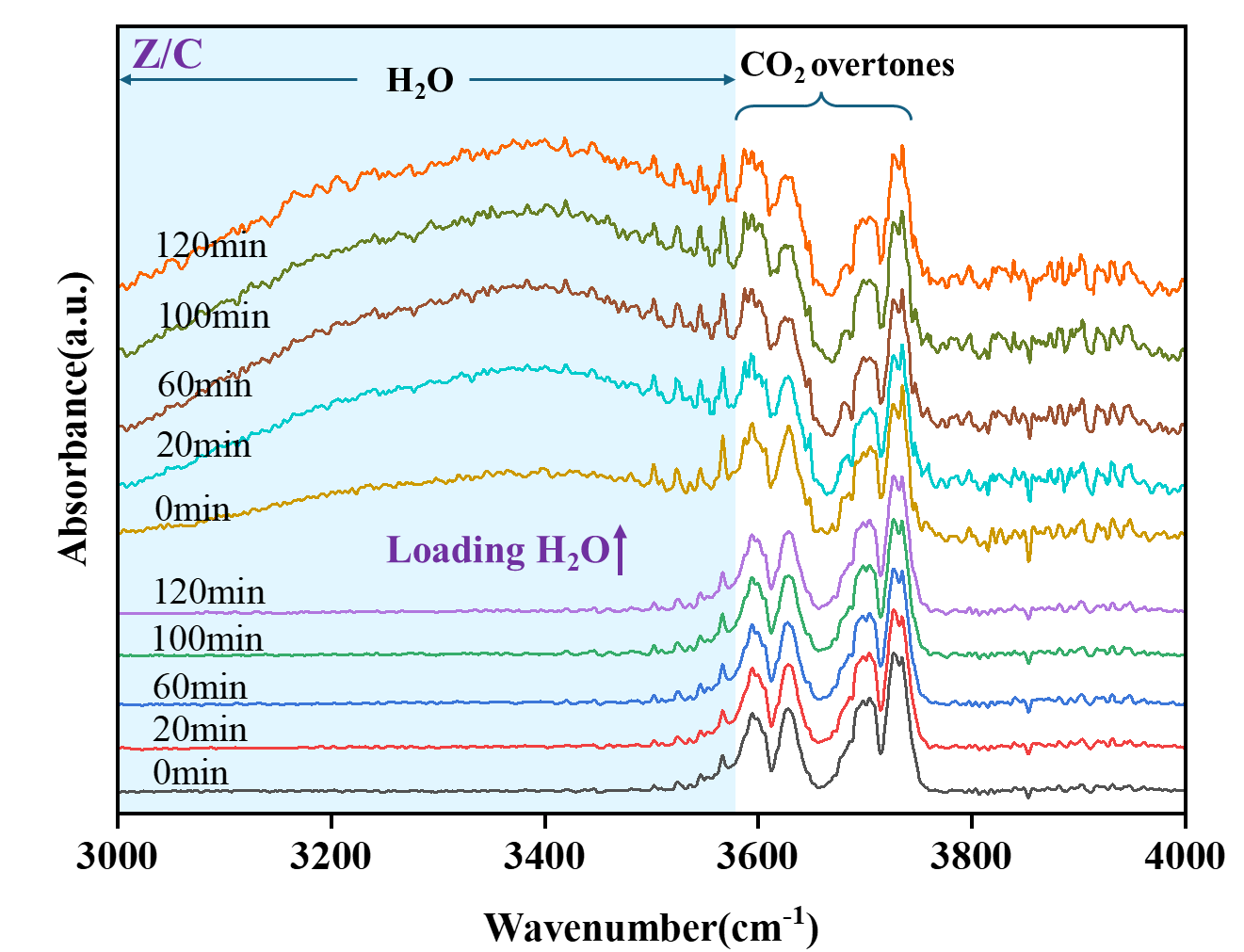


**Figure S22.** The in situ DRIFTS spectra of surface hydroxyl species over Z/C catalyst during CO_2_ hydrogenation at 240 °C with and without water.

**Table S1.** The physicochemical properties of different catalysts

| Catalysts |  | Zr loading^a^  (wt%) | Cu  loading^a^  (wt%) | Cu:Zr^a^  (molar ratio) | S_BET_^b^  (m^2^/g) | Vpore^b^  (cm^3^/g) | d_CuO_^c^  (nm) |
| --- | --- | --- | --- | --- | --- | --- | --- |
| Z/C |  | 13.4 | 86.6 | 9.3 | 26.43 | 0.154 | 11.83 |
| Z/C-P(1:0.5) |  | - | - | - | 14.10 | 0.132 | 11.65 |
| Z/C-P(1:1) |  | 11.2 | 88.7 | 11.4 | 13.08 | 0.075 | 11.34 |
| Z/C-P(1:2) |  | - | - | - | 7.04 | 0.031 | 11.62 |
| Z/C-P(1:5) |  | - | - | - | 6.26 | 0.029 | 11.47 |

1. The concentration of Cu and Zr was measured by ICP-OES. wt(Zr)% = C_ICP_(Zr) /(C_ICP_(Zr)+ C_ICP_(Cu)), wt(Cu)% = C_ICP_(Cu) /(C_ICP_(Zr)+ C_ICP_(Cu)).
2. Determined by BET method.
3. Determined by Scherrer equation from XRD patterns.

**Table S2.** The performance comparison of the modified catalyst and unmodified catalyst.

| Catalysts | CO_2_  conversion(%) | Selectivity(%) | | | STY_CH3OH_  (mg_CH3OH_ g_cat_^-1^ h^-1^) |
| --- | --- | --- | --- | --- | --- |
|  |  | MeOH | CO | CH4 |  |
| Z/C-P(1:1) | 8.5 | 65.78 | 33.94 | 0.28 | 920.10 |
| Z/C | 5.11 | 83.95 | 15.79 | 0.26 | 707.27 |

Reaction conditions: 240 ℃, 5 MPa, 48000 mL g_cat_^-1^ h^-1^, H_2_/CO_2_/Ar at 72/24/4 molar ratio.

**Table S3.** The catalytic performances of different catalysts in this work at 240 ℃

| Catalysts | CO_2_  conversion(%) | Selectivity(%) | | | STY_CH3OH_  (mg_CH3OH_ g_cat_^-1^ h^-1^) |
| --- | --- | --- | --- | --- | --- |
|  |  | CH_3_OH | CO | CH4 |  |
| Z/C | 5.11 | 83.95 | 15.79 | 0.26 | 707.27 |
| Z/C-P(1:0.5) | 7.37 | 66.81 | 32.89 | 0.30 | 879.35 |
| Z/C-P(1:1) | 8.5 | 65.78 | 33.94 | 0.28 | 920.10 |
| Z/C-P(1:2) | 8.85 | 63.82 | 36.00 | 0.18 | 929.51 |
| Z/C-P(1:5) | 8.93 | 63.35 | 36.50 | 0.15 | 931.01 |

a. Reaction conditions: 240 ℃, 5 MPa, 48000 mL g_cat_^-1^ h^-1^, H_2_/CO_2_/Ar at 72/24/4 molar ratio.

b. In the calculation of STY_CH3OH_, the value of catalyst mass only considered the amount of Z/C, and did not include the mass of PDVB.

**Table S4.** The catalytic performances of different catalysts in this work at 220 ℃.

| Catalysts | CO_2_  conversion(%) | Selectivity(%) | | | STY_CH3OH_  (mg_CH3OH_ g_cat_^-1^ h^-1^) |
| --- | --- | --- | --- | --- | --- |
|  |  | CH_3_OH | CO | CH4 |  |
| Z/C | 3.65 | 90.45 | 9.32 | 0.23 | 543.92 |
| Z/C-P(1:0.5) | 4.67 | 84.02 | 15.69 | 0.29 | 645.73 |
| Z/C-P(1:1) | 5.38 | 82.55 | 17.20 | 0.25 | 730.86 |
| Z/C-P(1:2) | 5.47 | 81.32 | 18.52 | 0.16 | 732.05 |
| Z/C-P(1:5) | 5.96 | 74.95 | 24.92 | 0.13 | 735.14 |

a. Reaction conditions: 220 ℃, 5 MPa, 48000 mL g_cat_^-1^ h^-1^, H_2_/CO_2_/Ar at 72/24/4 molar ratio.

b. In the calculation of STY_CH3OH_, the value of catalyst mass only considered the amount of Z / C, and did not include the mass of PDVB.

**Table S5.** Catalytic performances of as-synthesized and reported catalysts in similar conditions.

| No. | Catalyst | T  (℃) | P  (MPa) | H_2_:  CO_2_ | GHSV  (mL g_cat_^-1^ h^-1^) | Conv_CO2_ (%) | STY_CH3OH_  (mg g_cat_^-1^ h^-1^) | Ref |
| --- | --- | --- | --- | --- | --- | --- | --- | --- |
| 1 | FL-MoS_2_ | 240 | 5 | 3 | 36000 | 6.3 | 740 | ^[3]^ |
| 2 | In_2_O_3_ | 300 | 5 | 3 | 20000 | 5.2 | 295 | ^[4]^ |
| 3 | Cu/ZnO/MOF | 250 | 4 | 3 | 18000 | 3.3 | 173 | ^[5]^ |
| 4 | ZnZrOx/Cu | 220 | 5 | 3 | 48000 | 8.7 | 1045 | ^[1]^ |
| 5 | Commercial-Cu/ZnO/Al_2_O_3_ | 240 | 3 | 3 | 48000 | 7.2 | 771 | ^[6]^ |
| 6 | Cu-ZnO-ZrO_2_ | 240 | 3 | 3 | 39600 | 9.5 | 624 | ^[7]^ |
| 7 | ZnO-CuO-CP | 240 | 3 | 3 | 48000 | 4.8 | 432.9 | ^[6]^ |
| 8 | Cu/ZnO-CC | 240 | 3 | 3 | 42000 | 10.6 | 240 | ^[8]^ |
| 9 | Cu/ZnO/Al_2_O_3_ | 300 | 5 | 3 | 20000 | 2.1 | 122 | ^[9]^ |
| 10 | F-CuZn_553 | 220 | 4 | 3 | 56571 | 1.9 | 660 | ^[10]^ |
| 11 | ZrO_2_/Cu | 220 | 3 | 3 | 48000 | 4.5 | 524 | ^[11]^ |
| 12 | Z/C-P (1:1) | 240 | 5 | 3 | 48000 | 8.5 | 920.1 | This work |

**Table S6.** O 1s spectral characteristics of the water-treated catalysts

| Catalysts | O_β_ | | O_γ_ | | |
| --- | --- | --- | --- | --- | --- |
|  | Binding energy(eV) | Proportion  (%) | | Binding energy(eV) | Proportion  (%) |
| the reduced Z/C | 531.7 | 38.90 | | 532.8 | 15.93 |
| the water-treated Z/C | 531.7 | 27.50 | | 532.8 | 37.46 |
| the water-treated Z/C-P (1:1) | 531.7 | 36.50 | | 532.8 | 16.05 |

**Table S7.** DRIFTS peak assignments of the surface species for the CO_2_+ H_2_ reaction on the Z/C and Z/C-P (1:1) catalysts.

| Peaks(cm-1) | Species | Ref |
| --- | --- | --- |
| 1319 | HCOO* | ^[12-14]^ |
| 1370 | HCOO* | ^[15, 16]^ |
| 1582 | HCOO* | ^[12, 17]^ |
| 1050 | CH_3_O* | ^[15, 18, 19]^ |
| 1484 | CH_3_O* | ^[20]^ |
| 1455 | *CO_3_ | ^[21]^ |
| 1487 | *CO_3_ | ^[22]^ |
| 2822 | CH_3_O* (ν_s_(CH_3_)) | ^[15, 23, 24]^ |
| 2878 | HCOO* | ^[24, 25]^ |
| 2927 | CH_3_O* (ν_as_(CH_3_)) | ^[23]^ |
| 2965 | HCOO* | ^[24]^ |
| 3590-3750 | CO_2_(g) overtones | ^[25, 26]^ |
| 2500-3800 | H_2_O | ^[26-28]^ |

**References**

[1] Y. Xu, Z. Gao, Y. Xu, X. Qin, X. Tang, Z. Xie, J. Zhang, C. Song, S. Yao, W. Zhou, Cu-supported nano-ZrZnOx as a highly active inverse catalyst for low temperature methanol synthesis from CO_2_ hydrogenation, Applied Catalysis B: Environment Energy Fuels. 344 (2024) 123656.

[2] X. Li, Y. Xia, Y. Xu, H. Li, J. Huang, J. Yao, H. Zhao, X. Gao, J. Yu, L. Guo, Optimizing interfacial interaction between Cu and metal oxides boosts methanol yield in CO_2_ hydrogenation, Research on Chemical Intermediates. 49 (2023) 3933-3950.

[3] J. Hu, L. Yu, J. Deng, Y. Wang, K. Cheng, C. Ma, Q. Zhang, W. Wen, S. Yu, Y. Pan, Sulfur vacancy-rich MoS2 as a catalyst for the hydrogenation of CO_2_ to methanol, Nature Catalysis. 4 (2021) 242-250.

[4] O. Martin, A. J. Martín, C. Mondelli, S. Mitchell, T. F. Segawa, R. Hauert, C. Drouilly, D. Curulla‐Ferré, J. Pérez‐Ramírez, Indium oxide as a superior catalyst for methanol synthesis by CO_2_ hydrogenation, Angewandte Chemie. 128 (2016) 6369-6373.

[5] B. An, J. Zhang, K. Cheng, P. Ji, C. Wang, W. Lin, Confinement of ultrasmall cu/zno x nanoparticles in metal–organic frameworks for selective methanol synthesis from catalytic hydrogenation of CO_2_, Journal of the American Chemical Society. 139 (2017) 3834-3840.

[6] X. Sun, Z. Liu, S. Qian, K. Nie, Z. Li, W. Wan, B. Yan, Optimizing CO_2_ hydrogenation to methanol by enriching ZnOx/Cu interfacial sites in Zn-dispersed HKUST-1 derived catalysts, Applied Catalysis B: Environment Energy Fuels. 373 (2025) 125334.

[7] F. Arena, G. Mezzatesta, G. Zafarana, G. Trunfio, F. Frusteri, L. Spadaro, How oxide carriers control the catalytic functionality of the Cu–ZnO system in the hydrogenation of CO_2_ to methanol, Catalysis today. 210 (2013) 39-46.

[8] H. Lei, R. Nie, G. Wu, Z. Hou, Hydrogenation of CO_2_ to CH3OH over Cu/ZnO catalysts with different ZnO morphology, Fuel. 154 (2015) 161-166.

[9] R. Gaikwad, A. Bansode, A. Urakawa, High-pressure advantages in stoichiometric hydrogenation of carbon dioxide to methanol, Journal of catalysis. 343 (2016) 127-132.

[10] V. Dybbert, S. M. Fehr, F. Klein, A. Schaadt, A. Hoffmann, E. Frei, E. Erdem, T. Ludwig, H. Hillebrecht, I. Krossing, Oxidative Fluorination of Cu/ZnO Methanol Catalysts, Angew Chem Int Ed Engl. 58 (2019) 12935-12939.

[11] C. Wu, L. Lin, J. Liu, J. Zhang, F. Zhang, T. Zhou, N. Rui, S. Yao, Y. Deng, F. Yang, Inverse ZrO2/Cu as a highly efficient methanol synthesis catalyst from CO_2_ hydrogenation, Nature Communications. 11 (2020) 5767.

[12] W. Wu, Y. Wang, L. Luo, M. Wang, Z. Li, Y. Chen, Z. Wang, J. Chai, Z. Cen, Y. Shi, CO_2_ Hydrogenation over copper/ZnO single‐atom catalysts: water‐promoted transient synthesis of methanol, Angewandte Chemie International Edition. 61 (2022) e202213024.

[13] Y. Chen, C. Zhang, D. Yao, O. M. Gazit, Z. Zhong, Generating Strong Metal–Support Interaction and Oxygen Vacancies in Cu/MgAlO x Catalysts by CO_2_ Treatment for Enhanced CO_2_ Hydrogenation to Methanol, ACS Applied Materials & Interfaces. 17 (2025) 3404-3417.

[14] X. Wang, Y. Liu, Z. Wang, C. Chen, Z. Song, Y. Xu, D. Liu, Enhancement of Cu–Zn Interaction via Improved Cu–Zn Contact: Promoting Zinc Migration for Active Site Formation, Industrial & Engineering Chemistry Research. 64 (2025) 14449-14458.

[15] J. Weigel, R. Koeppel, A. Baiker, A. Wokaun, Surface species in CO and CO_2_ hydrogenation over copper/zirconia: On the methanol synthesis mechanism, Langmuir. 12 (1996) 5319-5329.

[16] H. Chang, F. Gao, S. Ma, Y. Zhu, Z. Liu, J. Liu, H. He, K. Zhang, Y. Liu, Y. Cao, Unlocking Methanol Synthesis from CO_2_ and H2 on ZnO/ZrO2 Catalysts: Surface Hydroxyl-Mediated Activation, Acs Catalysis. 15 (2025) 6005-6017.

[17] J. Toyir, P. R. de la Piscina, N. Homs, Ga-promoted copper-based catalysts highly selective for methanol steam reforming to hydrogen; relation with the hydrogenation of CO_2_ to methanol, International Journal of Hydrogen Energy. 40 (2015) 11261-11266.

[18] X. Zhang, Z. Sun, Y. Shan, H. Pan, Y. Jin, Z. Zhu, L. Zhang, K. Li, Boosting methanol production via plasma catalytic CO 2 hydrogenation over a MnO x/ZrO 2 catalyst, Catalysis Science & Technology. 13 (2023) 2529-2539.

[19] Z. Wu, M. Li, D. R. Mullins, S. H. Overbury, Probing the surface sites of CeO2 nanocrystals with well-defined surface planes via methanol adsorption and desorption, Acs Catalysis. 2 (2012) 2224-2234.

[20] L. Huang, L. Cui, C. Liu, X. Wei, Y. Liu, F. Cao, Enhancement of CO 2 hydrogenation to methanol over Cu-based catalysts mixed with hydrophobic additives, Catalysis Science Technology. (2025).

[21] Y. Ma, J. Wang, K. R. Goodman, A. R. Head, X. Tong, D. J. Stacchiola, M. G. White, Reactivity of a zirconia–copper inverse catalyst for CO_2_ hydrogenation, The Journal of Physical Chemistry C. 124 (2020) 22158-22172.

[22] W. Xiong, J. Ding, D. Wang, W. Huang, Cu facet-dependent elementary surface reaction kinetics of CO_2_ hydrogenation to methanol catalyzed by ZrO_2_/Cu inverse catalysts, The Journal of Physical Chemistry Letters. 14 (2023) 7229-7234.

[23] F. C. Meunier, I. Dansette, K. Eng, Y. Schuurman, Differentiating the reactivity of ZrO2-bound formates formed on Cu/ZrO2 during CO_2_ hydrogenation, Catalysts. 12 (2022) 793.

[24] K.-D. Jung, A. T. Bell, Role of hydrogen spillover in methanol synthesis over Cu/ZrO2, Journal of catalysis. 193 (2000) 207-223.

[25] Y. Wang, S. Kattel, W. Gao, K. Li, P. Liu, J. G. Chen, H. Wang, Exploring the ternary interactions in Cu–ZnO–ZrO2 catalysts for efficient CO_2_ hydrogenation to methanol, Nature Communications. 10 (2019) 1166.

[26] J. C. Wu, C.-W. Huang, In situ DRIFTS study of photocatalytic CO_2_ reduction under UV irradiation, Frontiers of Chemical Engineering in China. 4 (2010) 120-126.

[27] J. Saussey, J. Lavalley, An in situ FT-IR study of adsorbed species on a Cu-ZnAl2O4 methanol catalyst under 1 MPa pressure and at 525 K: effect of the H2/CO/CO_2_ feed stream composition, Journal of molecular catalysis. 50 (1989) 343-353.

[28] A. V. Tarasov, F. Seitz, R. Schlögl, E. Frei, In situ quantification of reaction adsorbates in low-temperature methanol synthesis on a high-performance Cu/ZnO: Al catalyst, Acs Catalysis. 9 (2019) 5537-5544.
